# Towards Prebiotic Catalytic Amyloids Using High Throughput Screening

**DOI:** 10.1371/journal.pone.0143948

**Published:** 2015-12-09

**Authors:** Michael P. Friedmann, Vladimir Torbeev, Viviane Zelenay, Alexander Sobol, Jason Greenwald, Roland Riek

**Affiliations:** 1 Laboratory of Physical Chemistry, Department of Chemistry, ETH Zürich, Zurich, Switzerland; 2 Laboratory of Organic Chemistry, Department of Chemistry, ETH Zürich, Zurich, Switzerland; University of Maryland School of Medicine, UNITED STATES

## Abstract

Enzymes are capable of directing complex stereospecific transformations and of accelerating reaction rates many orders of magnitude. As even the simplest known enzymes comprise thousands of atoms, the question arises as to how such exquisite catalysts evolved. A logical predecessor would be shorter peptides, but they lack the defined structure and size that are apparently necessary for enzyme functions. However, some very short peptides are able to assemble into amyloids, thereby forming a well-defined tertiary structure called the cross-β-sheet, which bestows unique properties upon the peptides. We have hypothesized that amyloids could have been the catalytically active precursor to modern enzymes. To test this hypothesis, we designed an amyloid peptide library that could be screened for catalytic activity. Our approach, amenable to high-throughput methodologies, allowed us to find several peptides and peptide mixtures that form amyloids with esterase activity. These results indicate that amyloids, with their stability in a wide range of conditions and their potential as catalysts with low sequence specificity, would indeed be fitting precursors to modern enzymes. Furthermore, our approach can be efficiently expanded upon in library size, screening conditions, and target activity to yield novel amyloid catalysts with potential applications in aqueous-organic mixtures, at high temperature and in other extreme conditions that could be advantageous for industrial applications.

## Introduction

Unfortunately, there is no fossil or genetic record leading back to the time, more than 3.5 billion years ago, when sustainable life first appeared on our planet [1]. We can, therefore, only speculate about the details of this event. However, speculation by way of deduction may lead to some reasonable conclusions about the nature of the first replicative molecules. Such an approach resulted in the “RNA world” hypothesis in which RNA plays the central role as both the replicative and the functional element [2]. There are, however, many compelling arguments for other concepts on the origin of life such as the “protein first” [3,4] and the “amyloid world” [5,6] hypotheses (Reviews [7,8]). Amyloid fibrils are highly ordered protein or peptide aggregates whose substructure consists of a repetitive pattern of intermolecular β-strands that run perpendicular to the fibrillar axis, forming what is called a cross-β-sheet. Thus, amyloids are composed of an ordered arrangement of many copies of a peptide forming a 1D crystal-like protein fold [9]. The idea that peptides existed on a primitive earth appears reasonable given the abundant evidence of amino acids on meteorites as well as the known prebiotic mechanisms for both synthesis of amino acids [10] and condensation of amino acids into peptides [11–14]. Since short peptides of 4 to 6 amino acid residues can form amyloids [9], peptide amyloids have the potential to be among the first prebiotic replicative systems. One of the first selection criteria could have been the inherent stability of amyloids [7], which protected the peptides that could form amyloids in the harsh prebiotic environment of intense UV irradiation, large temperature and pH fluctuations as well as high salt concentration [15–18]. Further arguments to support the idea that protein aggregation has played a key role in the early evolution of life are reviewed in [8].

Interestingly however, there is still little known about the ability of amyloids to perform the functions of enzymes. Macromolecular β-sheet-rich structures formed from basic peptides with simple repetitive sequences have been shown to have phosphodiesterase activity [19,20] and amyloids have been shown to template their own synthesis from two activated peptide precursors [21,22]. With these promising indications, we set out to address the question whether one can systematically explore the catalytic potential of amyloids? To this end, we have applied a library-based screening approach to search for amyloid catalysts. The most active catalytic amyloids that we found in this way recapitulate the recent findings by Rufo *et al*. [23] that a catalytic amyloid can be designed to mimic the structure of an enzyme active site.

## Results and Discussion

### Technical aspects of an amyloid library

While there are many well developed techniques that have been applied to the construction of protein and peptide libraries [24–26], amyloids present several unique challenges that render the traditional approaches largely irrelevant. Of primary concern is that amyloids do not exist as soluble homogeneous species but rather as large, mostly insoluble assemblies of varying aggregation number. Therefore, quantitative routine handling of a diverse amyloid library may be difficult due to the variable behavior of amyloids in solution, ranging from flocculent precipitates to hydrated gels.

The traditional libraries (*i*.*e*. phage display, mRNA display, or one-bead one-peptide) do not permit the spatial and conformational freedom required for the aggregation of a large number of peptides. Recombinant expression in *E*. *coli* is precluded by the fact that short peptides are generally not stably expressed except as fusions to larger proteins. One elegant solution for a gene-based amyloid library involves the concatenation of several aggregating segments into larger proteins and expressing them in *E*. *coli* [27]. The size and complexity of these concatenated constructs, however, lie outside the scope of an investigation into short prebiotic peptides.

Our solution to these challenges was to create a library of short synthetic peptides using a parallel peptide synthesizer. The peptides have been designed to contain a common amyloidogenic motif (see below) with the goal of ensuring a consistent aggregation. The strong tendency of these peptides to aggregate permitted the production of the amyloid library without purification, using fibrils of the crude solid phase peptide synthesis (SPPS) reaction products. The lyophilized reaction products were solubilized at a peptide concentration of 4 mM in either a solution of 99% DMSO with 1% TFA or a solution of 10 mM NaOH in 90% DMSO and 10% H_2_O. Fibrillization was initiated by diluting the solubilized peptides (and peptide mixtures) 25 times into the desired fibrillization condition (*e*.*g*. 50 mM HEPES, pH 7.3) yielding a peptide concentration of 160 μM. With this approach an amyloid library was created that could easily be scaled-up to take advantage of high-throughput screening (HTS) methodologies; that is, the number of steps required from library production to screening was minimized, and universally applicable handling procedures were established with no special treatment given to individuals of the library. As a proof of this amyloid library concept, we chose a library size of 94 amyloids (see below) that was large enough to employ HT methodologies yet still manageable in a mostly non-HTS setting.

### Physicochemical aspects of an amyloid library

A high β-aggregation propensity is essential for a combinatorial peptide library in the form of amyloids. To satisfy this requirement we profited from the well-documented observation that in aqueous environments, binary alternating sequences of hydrophobic-hydrophilic residues are prone to oligomerize into amphipathic β-structures. In these structures, the hydrophobic residues are buried at the interface of two β-sheets, leaving the hydrophilic residues on the surface [28,29]. In keeping with the theme of a prebiotic fold, we wanted to use the shortest peptides that still guaranteed a high percentage of amyloidogenic peptides in the library. To this end, we made a series of C-terminally amidated but not acetylated peptides of increasing length with sequences of alternating Val and Ser from 4 to 9-mers (Table 1). The CD spectra of these peptides in 50 mM HEPES buffer pH 7.3 after fibrillization for 2 days revealed that a minimum of three Val residues was required for β-structure and that the peptide V**S**V**S**V**S**V-CONH_2_ with four Val residues was essentially completely aggregated at 200 μM since less than 5% of the peptide was in solution after centrifugation at 25,000 g. Furthermore, the peptides with three or four Val residues retained their β-structure also at pH 4.3 in 50 mM NaOAc as monitored by CD spectroscopy, despite the predicted net charge at more than 3 pH units below the p*K*_a_ of the N-terminus. In fact the four Val peptides were so aggregation prone that purification by reversed-phase chromatography was challenging. It is noteworthy, that despite the significant contaminants of deletions, truncations and a C-terminal *p*-hydroxybenzyl side-product [30], the crude peptides were fully capable of forming amyloids.

**Table 1:** Solubility and secondary structure of binary alternating peptides.

Sequence	2° structure^1^	Soluble^2^	Purity
V**S**V**S**-CONH_2_	--^3^	1.0	crude / ~60%
**S**V**S**V**S**-CONH_2_	--	1.0	crude / ~60%
V**S**V**S**V**S**-CONH_2_	--	1.0	crude / ~60%
**S**V**S**V**S**V**S**-CONH_2_	rc/β	0.63	crude / ~50%
V**S**V**S**V**S**V**S**-CONH_2_	rc/β	--^4^	crude / ~50%
**S**V**S**V**S**V**S**V**S**-CONH_2_	β	0.38	crude / ~50%
**S**V**S**V-CONH_2_	--	1.0	HPLC / >95%
V**S**V**S**V-CONH_2_	--	1.0	HPLC / >95%
**S**V**S**V**S**V-CONH_2_	β	0.38	HPLC / >95%
V**S**V**S**V**S**V-CONH_2_	β	0.05	HPLC / >95%

^1^ The secondary structure was estimated from the CD spectrum of the precipitate derived from a 0.2 mg/ml solution of peptide in 50 mM phosphate buffer pH 8. When the precipitate was transferred to the pH 4 buffer, the spectra were essentially the same.

^2^ The amount of the peptide in the supernatant and precipitate fractions was determined by hydrolysis (see Materials and Methods).

^3^ The CD spectra of samples indicated had no sufficient precipitate to be measured.

^4^ The quantitation of this sample was prevented by an interfering compound (HPLC).

Based on these results we chose to construct a library of unpurified octapeptides from the template Ac-YV**S**V**S**V**S**V-CONH_2_ in which Ser was replaced with Asp, His and Ala. The residues Asp and His were chosen for their potential role as a general acid or base in catalysis, as well as for their contrasting p*K*_a_. The substitutions with Ala were included to modulate the hydrophobicity of the polar face of the amyloid and the Tyr residue was included to aid in quantitation. Of the 64 possible combinations of four amino acids in three positions, we made a selection of 33 peptides (Table 2). We further expanded the library to 94 amyloids by non-systematically combining the 33 peptides to generate 61 binary peptide mixtures (Figure A in S1 File). We reasoned that the co-aggregation into mixed peptide amyloids is realistic given the fact that it has been observed in nature as well as in designed peptides [31,32]. The resulting library of 94 amyloids, with the inclusion of a positive and negative control, was subsequently fibrillized under a variety of conditions and prepared for activity screening in a 96-well format (see Materials and Methods).

**Table 2:** Peptides used in this study.

Peptide	Composition
1	Ac-YV**D**V**D**V**D**V-CONH_2_
2	Ac-YV**S**V**D**V**D**V-CONH_2_
3	Ac-YV**D**V**S**V**D**V-CONH_2_
4	Ac-YV**D**V**D**V**S**V-CONH_2_
5	Ac-YV**S**V**S**V**D**V-CONH_2_
6	Ac-YV**S**V**D**V**S**V-CONH_2_
7	Ac-YV**D**V**S**V**S**V-CONH_2_
8	Ac-YV**H**V**H**V**H**V-CONH_2_
9	Ac-YV**S**V**H**V**H**V-CONH_2_
10	Ac-YV**H**V**S**V**H**V-CONH_2_
11	Ac-YV**H**V**H**V**S**V-CONH_2_
12	Ac-YV**S**V**S**V**H**V-CONH_2_
13	Ac-YV**S**V**H**V**S**V-CONH_2_
14	Ac-YV**H**V**S**V**S**V-CONH_2_
15	Ac-YV**D**V**H**V**S**V-CONH_2_
16	Ac-YV**D**V**S**V**H**V-CONH_2_
17	Ac-YV**H**V**D**V**S**V-CONH_2_
18	Ac-YV**S**V**D**V**H**V-CONH_2_
19	Ac-YV**S**V**H**V**D**V-CONH_2_
20	Ac-YV**H**V**S**V**D**V-CONH_2_
21	Ac-YV**H**V**H**V**D**V-CONH_2_
22	Ac-YV**H**V**D**V**H**V-CONH_2_
23	Ac-YV**D**V**H**V**H**V-CONH_2_
24	Ac-YV**D**V**D**V**H**V-CONH_2_
25	Ac-YV**D**V**H**V**D**V-CONH_2_
26	Ac-YV**H**V**D**V**D**V-CONH_2_
27	Ac-YV**A**V**H**V**H**V-CONH_2_
28	Ac-YV**H**V**A**V**H**V-CONH_2_
29	Ac-YV**D**V**H**V**A**V-CONH_2_
30	Ac-YV**A**V**D**V**H**V-CONH_2_
31	Ac-YV**H**V**D**V**A**V-CONH_2_
32	Ac-YV**H**V**A**V**D**V-CONH_2_
33	Ac-YV**A**V**H**V**D**V-CONH_2_
34	Ac-I**H**I**H**I**Q**I-CONH_2_
35	Ac-YV**H**V**H**V**A**V-CONH_2_
36	Ac-**H**G**H**-CONH_2_

### Characterization of amyloid library and extent of aggregation

The concept of creating an amyloid library out of a peptide library is only meaningful if the vast majority of the peptides actually form amyloids in at least one of the conditions tested. To establish the validity of our amyloid library we measured circular dichroism (CD), Fourier transform infrared spectroscopy (ATR-FTIR), as well as thioflavin T (ThT) fluorescence of peptides **1**–**33** at pH 7.3 (50 mM HEPES) and pH 4 (50 mM NaOAc) (see interactive Table A and associated data in S1 File). Based on the analysis of the CD and ATR-FTIR spectra (shown in the interactive Table A in S1 File) all the peptides formed β-structured aggregates in at least one condition. Most of the 33 peptides induced a significant increase in ThT fluorescence in at least one condition. Furthermore, all 33 peptides were found to form amyloid-like fibrils in at least one condition as observed by TEM. The fibrils from a selection of the active peptides were aligned and analyzed by X-ray diffraction (Figures C and D in S1 File). In each case we observed the cross-β diffraction pattern typical of amyloid fibrils, although the degree of sample alignment varied. For two samples, **14** and **27**, the difference in meridional and equatorial diffraction intensities was small but easily detected in a plot of the angular dependence of the intensity (Figure D in S1 File). The conclusion that can be drawn out of the multiple analysis techniques is that the 33 peptides that comprise the library are overwhelmingly prone to form fibril-like, β-structured aggregates. The cross-β diffraction of the tested aggregates would imply that they are more specifically all amyloidogenic.

### Zinc(II)-dependent hydrolytic activity of amyloids at pH 7.3

**Fig 1:**
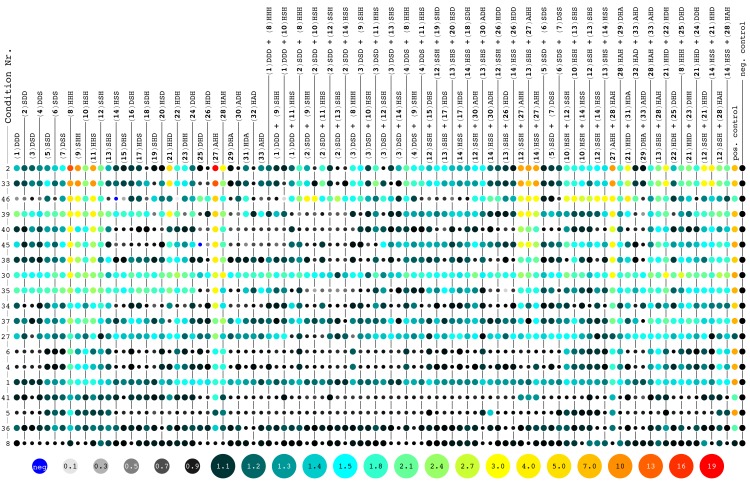
Activity screens of peptide amyloids in various conditions. The hydrolysis rates of 4NPA measured for each of the peptide amyloids (in columns) are shown as a ratio to the negative control (buffer) with color-codes as indicated at the bottom of the Figure. A color code from black (rate ratio of 1.0) to light blue (1.5) to red (19) is used. Activities below the negative control are indicated with smaller disks and color-coded from light grey to black. Occasionally observed negative reaction rates are indicated by small blue disks. The peptide amyloids were tested in a total of 46 conditions of which 19 conditions are shown here (in rows). The peptides are numbered according to Table 2, including a short nomenclature highlighting only the variable residues (*e*.*g*. the peptide Ac-YV**D**V**H**V**S**V-CONH_2_ is abbreviated DHS). The conditions are sorted top-down with the highest observed activity at the top. A detailed list of the conditions tested are shown in Table B in S1 File, while below a compressed introduction to the different conditions is listed: (2) pH 7.3 with ZnCl_2_ (control for 33 without heating), (33) condition 2 and heated to 95°C for 1 h prior to screening, (46) condition 2 and screened in 50% DMSO, (39) condition 2 and screened with 4.75 M NaCl, (40) condition 2 and screened with 0.2 M MgCl_2_, (45) condition 2 and screened in 90% EtOH, (38) condition 2 and screened with 0.2 M NaCl, (30) pH 7.3 with ZnCl_2_ and 1 M Na_2_SO_4_, (35) pH 7.3 and screened in 4.75 M NaCl, (34) pH 7.3 and screened in 0.5 M NaCl, (37) pH 7.3 and screened in 2 M MgCl_2_, (27) pH 7.3 with ZnCl_2_ and 0.2 M MgCl_2_, (6) pH 7.3 with NiCl_2_, (4) pH 7.3 with CoCl_2_, (1) pH 7.3 without other additives, (41) condition 2 and screened in 2 M MgCl2, (5) pH 7.3 with CuCl_2_, (36) pH 7.3 and screened in 0.2 M MgCl_2_ and (8) pH 4.0 without other additives.

The most common assay for hydrolytic activity in enzymes is with the chromogenic substrate *p*-nitrophenyl acetate (4NPA). Therefore, 4NPA was chosen permitting a direct comparison of the hits from the screen with natural enzymes. Using an absorbance plate reader, we screened the activity of the amyloid library at a peptide concentration of 40 μM, fibrillized at pH 4 or 7.3 and in the presence of divalent cations (250 μM Co^2+^, Cu^2+^, Mg^2+^, Mn^2+^, Ni^2+^, or Zn^2+^) (Fig 1). Each plate had a positive control of 200 μM imidazole (second to last column in Fig 1) and a negative control of buffer alone (last column in Fig 1). Imidazole is known to catalyze 4NPA hydrolysis under these conditions via an acyl intermediate [33] and it was used as a standard to compare the measured activity of the peptides from plate to plate.

**Fig 2:**
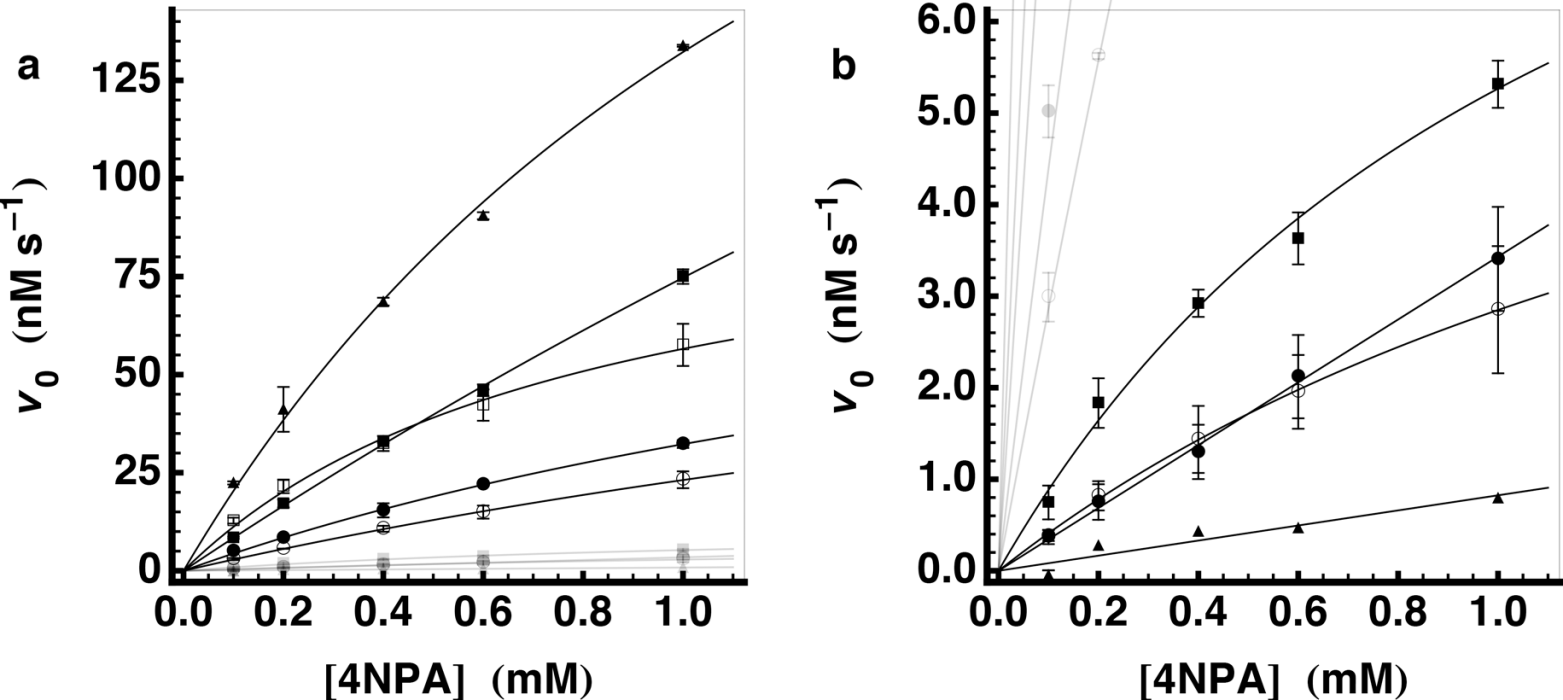
Esterase activity of selected peptides at pH 7.3. Esterase activity for a selection of peptides is shown. Plots show the initial hydrolysis rate of 4NPA as a function of initial substrate concentration together with the fit of the data to the Michaelis-Menten equation. The symbols for the individual peptides are in Table 3. In **(a)** all of the peptides that were measured are shown (the less active peptides greyed out), while in **(b)** the same data is plotted on a smaller scale to show the less active peptide amyloids.

Several amyloids at pH 7.3 with zinc(II) had a hydrolytic activity that was significantly above background (Fig 1, first row). The highest activity was seen for **8**, **9**, **11** and **27**, all of which share the HXH sequence motif that was recently reported by Rufo *et al*. to form catalytic amyloids [23]. We selected a few of the hits from the screen to measure the kinetic parameters of the amyloids of the purified peptides. At pH 7.3, the highest *k*_cat_ measured was 3.1 ± 0.8 × 10^-2^ s^-1^ (**27**, Ac-YV**A**V**H**V**H**V-CONH_2_). A value being comparable to the highest *k*_cat_ reported by Rufo *et al*. (Ac-I**H**I**H**I**Q**I-CONH_2_). To facilitate a direct comparison of these two results we synthesized and purified the latter peptide (our peptide **34**, Rufo peptide **11**). The kinetic parameters for the amyloids of these two peptides were very similar (Table 3 and Fig 2). Based on the activity of **27**, we synthesized its isomer (**35**, Ac-YV**H**V**H**V**A**V-CONH_2_).

**Table 3:** Esterase activity of selected peptides at pH 7.3.

	Composition	Symbol^1^	*k*_cat_/*K*_M_ (M^-1^s^-1^)	*k*_cat_ (10^-2^s^-1^)	*K*_M_ (mM)
8^2^	Ac-YV**H**V**H**V**H**V-CONH2	●	2.288±0.077	0.547±0.056	2.39±0.32
9	Ac-YV**S**V**H**V**H**V-CONH2	○	1.461±0.094	0.544±0.048	3.71±0.39
11	Ac-YV**H**V**H**V**S**V-CONH2	□	6.29±0.52	0.514±0.051	0.87±0.15
27^2^	Ac-YV**A**V**H**V**H**V-CONH2	■	4.26±0.13	3.05±0.80	7.2±2.1
34^2^	Ac--I**H**I**H**I**Q**I-CONH2	▲	15.76±0.96	2.47±0.32	1.57±0.29
35^2^	Ac-YV**H**V**H**V**A**V-CONH2		7.38±0.63	0.463±0.039	0.63±0.10
10	Ac-YV**H**V**S**V**H**V-CONH2	●	0.172±0.003^4^		
21	Ac-YV**H**V**H**V**D**V-CONH2	■	0.473±0.046	0.059±0.010	1.23±0.32
28	Ac-YV**H**V**A**V**H**V-CONH2	○	0.215±0.007	0.042±0.004	1.95±0.23
36^3^	Ac---**H**G**H**----CONH2	▲	0.041±0.005^4^		
	Imidazole		0.199±0.008^4^		

^1^ Symbol used in Fig 2A (first 5) and 2b (last 4).

^2^ Measurements taken in duplicate for these peptides, otherwise in triplicate.

^3^ Measurements taken without replicates.

^4^ For these *k*_cat_/*K*_M_ = *k*_2_ was obtained through a linear fit to *v*_0_ = [E]_0_ *k*_2_[S].

The comparison of **9** to **11** and **27** to **35** indicates that the sequence position of the HXH motif is not crucial for the appearance of the hydrolytic activity. Whereas *k*_cat_ varies only slightly (except for **27**), *K*_M_ shows a dependence on sequence position Better binding of the substrate in these cases occurs when the HXH motif is closer to the N-terminus, which might be explained by an interaction of the aromatic substrate with the aromatic Tyr residue. Furthermore, the identity of the surface-exposed amino acid adjacent to the HXH motif can be of importance as shown by **21** for which the neighboring Asp residue reduces the *k*_cat_ by one order of magnitude compared to the Ala and Ser analogues. The spacing between the His side chains is also important, as separating the two His residues of the HXH motif to HXXXH (**28**) similarly reduces the *k*_cat_.

### Structure-activity relationship of peptide amyloid catalysts

For the hydrolytically active peptides identified above, we found that the only measureable activity was in the insoluble fraction. The supernatant after centrifugation at 100,000 g had no detectable activity and the pellet retained comparable activity to the original sample. These findings indicated that the amyloid structure is the catalytic active state of the peptide. It was not possible to directly measure the monomer activity because the active peptides all aggregated within seconds of being transferred to the assay conditions. In order to assess the possibility that the soluble peptides are also catalytically active we synthesized the tripeptide Ac-**H**G**H**-CONH_2_ (**36**), a soluble variant of the HXH motif. We found that the 4NPA hydrolysis activity of **36** was approximately two orders of magnitude lower than the HXH-containing amyloids or only 0.3 times the background hydroxide catalyzed rate. It is approximately three times less active than imidazole and it is not zinc(II)-dependent. This comparison further supports the hypothesis that the amyloid is the functional state of the peptides.

**Fig 3:**
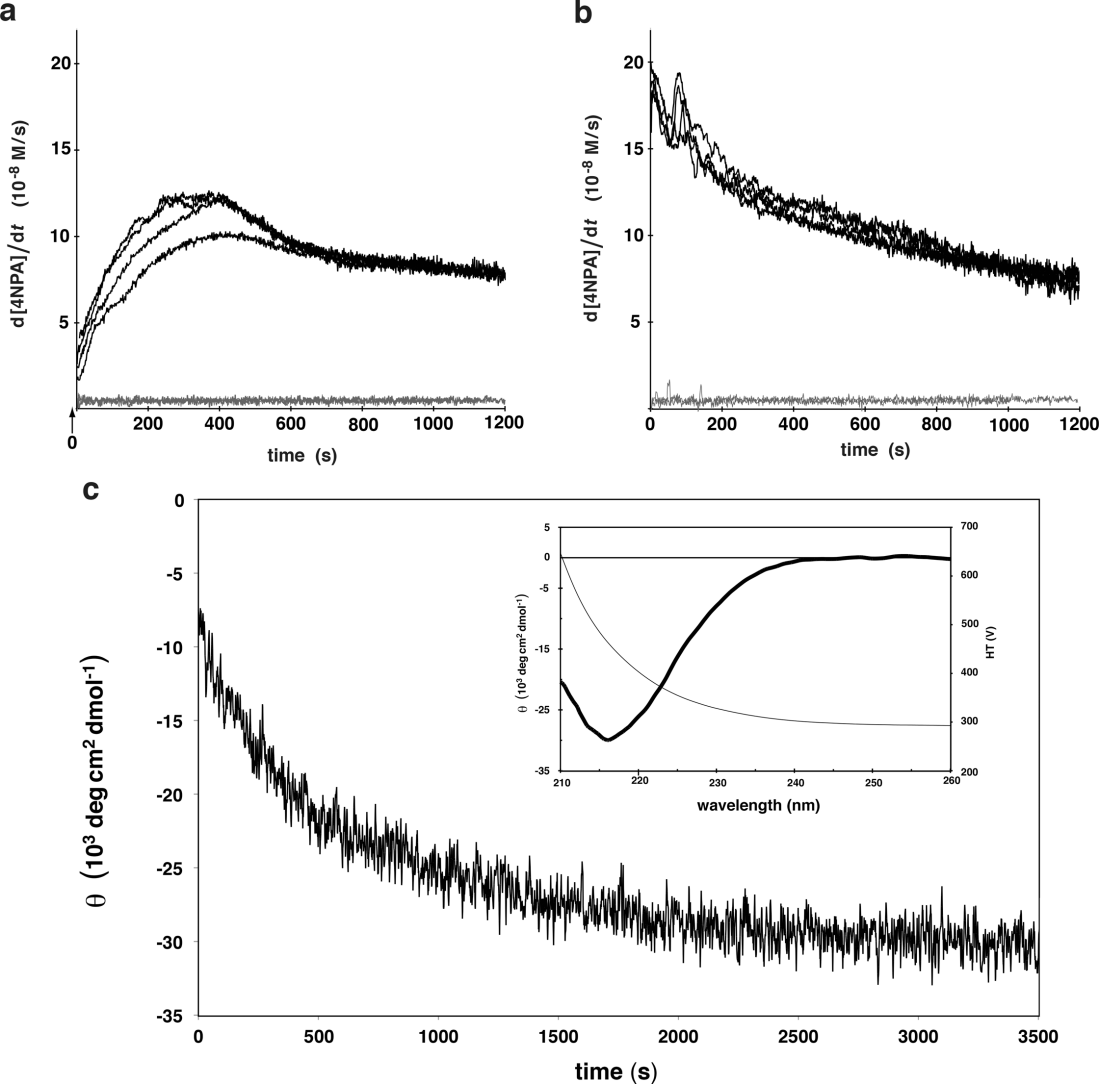
Parallel kinetics of fibrillization and hydrolysis activity. Kinetics of fibrillization measured by CD **(c)** matches the appearance of the 4NPA hydrolysis activity **(a). a)** A stock of **35** at 200 μM in 10 mM HCl was diluted to a final concentration of 50 μM into 50 mM HEPES pH 7.3 with 0.5 mM ZnCl_2_ and 1 mM 4NPA. After ca. 10 s mixing time, the hydrolysis of 4NPA was recorded every 1 s for 20 min at 400 nm and the first derivative of this signal (the instantaneous rate) is plotted as a function of time. The black lines are four independent measurements of these and the grey lines are two measurements of the background reaction (*i*.*e*. without peptide). **b)** Aggregates of **35** were prepared as in **(a)** except in the absence of substrate. After 1 h, 1 mM 4NPA was added and the hydrolysis rate was monitored. Once again, the black curves are 4 separate measurements and the grey lines 2 background measurements. **c)** Peptide **35** was prepared as in **(a)** and the CD signal at 218 nm was measured every 2 s starting ca. 12 s after mixing. The inset plot shows the CD spectrum of the sample after 1 h and the detector voltage (scale on the right).

For more direct evidence that the amyloid is the catalytically active state, we attempted to measure the catalysis rate during the aggregation of **35** in order to correlate the amyloid formation with an increase in hydrolytic activity. Since the aggregation is too fast to measure a steady-state hydrolysis rate, an instantaneous time-dependent rate of 4NPA hydrolysis was calculated from the derivative of the A_400_ measurements (Fig 3A). In parallel, an identical sample was monitored at 218 nm for the β-strand-typical ellipticity (Fig 3C). Note that the absorbance of 50 mM HEPES prevented the measurement of a full spectrum (190–260 nm) under the identical aggregation conditions, however when performed with 10 mM HEPES, the spectrum clearly indicates a move towards increasing β-structure upon mixing the peptide with buffer at pH 7.3 (Figure B in S1 File). Considering that the measurements had a dead time of 10 s from the time of mixing to the start of the measurement, it appears that the onset of hydrolysis activity begins close to *t* = 0 s, the moment that the peptide was transferred into the fibrillization buffer. To exclude any effect of sample mixing, the measurement was also performed in the same conditions but instead of mixing the peptide with the substrate at the time of fibrillization, the substrate was added 1 h later. In this case the reaction rate is maximal at t = 0 s (Fig 3B). Thus by extrapolation it was observed that the activity of the monomeric peptide is not significantly above the background hydrolysis (Fig 3A). Since the rate at which the hydrolysis activity evolved in the aggregating samples is similar to the kinetics of β-structure formation (Fig 3C) we conclude that the amyloid state and not the monomeric form of the peptides is the catalytically active state.

### Hydrolysis activity of amyloids under various extreme conditions

The general stability of the amyloid fold and the recognized amyloid structure-activity relationship let us explore the catalytic activity of amyloids under various and in part extreme conditions. To do so the fibrillization growth and the hydrolysis assay were performed under conditions of extreme salt concentration (up to 4.75 M NaCl), temperature (up to 95°C), pH (down to pH 2.2) as well as replacing water with other solvents (up to 90% ethanol and 50% DMSO) (Table B in S1 File). The 46 conditions expanded the library by 94 × 46 = 4324 amyloids in different conditions of which 19 are shown in Fig 1.

While, none of the new conditions yielded a more efficient catalyst, the activity of **27** and other HXH motif-containing peptide amyloids were often significant, including conditions with high organic solvent content (Fig 1, condition 45 with 50% DMSO and condition 46 with 90% EtOH) or high salt concentrations (Fig 1, condition 39 with 4.75 M NaCl and condition 30 with 1 M Na_2_SO_4_). Furthermore, incubating the samples at 95°C for 1 h prior to measurement did not significantly perturb the activity (Fig 1, condition 33).

It is interesting to note that in 50% DMSO with zinc(II) (Fig 1, condition 46) a series of amyloids composed of peptide mixtures (*e*.*g*. **2**/**9**, **2**/**10**, and **12**/**14**) but only one single peptide amyloid (*i*.*e*. **25**) showed both an increased relative activity compared to the same condition without DMSO (condition 2, top row in Fig 1) and activity above the corresponding positive control. While many of these amyloids still have peptides with the HXH motif, there are a number of individuals with only a single His per peptide sequence (*e*.*g*. **25**, **12**/**13**, **12**/**14**, and **13**/**14**). These findings indicate that under some environmental conditions sequences with a single His per peptide are becoming competitive against the HXH motif.

In the absence of ZnCl_2_ the otherwise active amyloids generally lost their activity to a point below the one of the positive control (Fig 1, conditions 1, 2, 4, 5, 8, 34, 35, 36, 37). A finding that had to be expected following the proposed mechanism of action by Rufo *et al*. [23].

### Metal-independent activity of mixed peptide amyloids at pH 4

**Fig 4:**
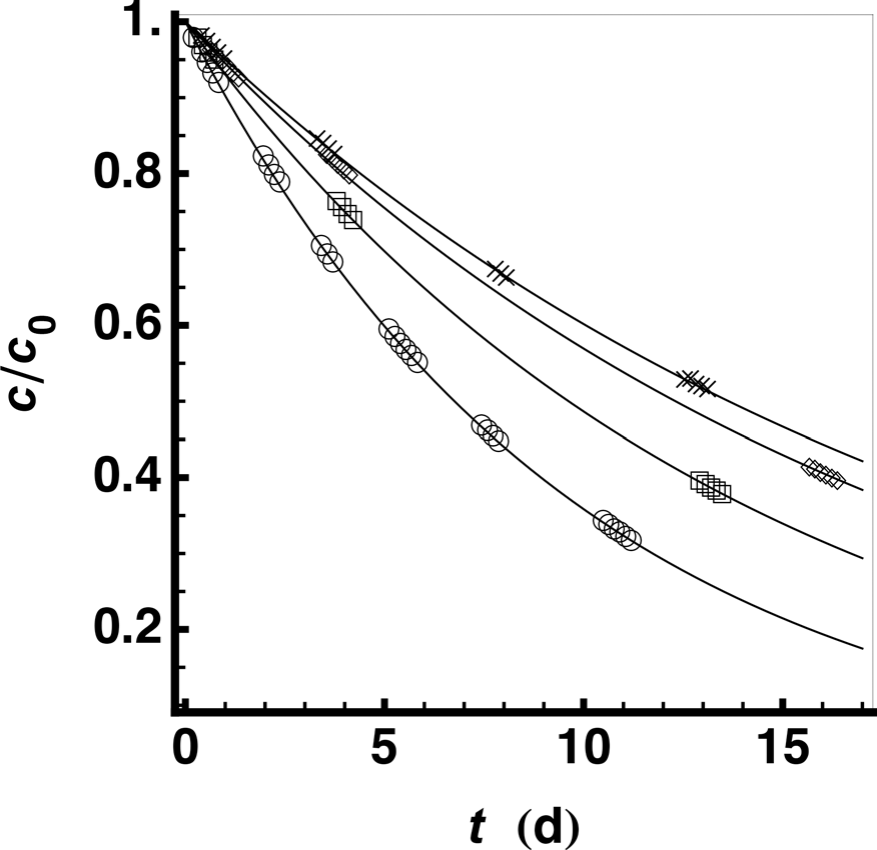
Esterase activity at pH 4.3 measured by NMR. The relative concentration of substrate measured by 1D-^1^H NMR peak intensities is shown as a function of reaction time for: (◇) 40 μM of **21**; (□) 40 μM of **12** and; (○) co-aggregate of 20 μM of **12** with 20 μM of **21**; (×) buffer control (50 mM d_3_-NaOAc at pH 4.3 and 2% d_6_-DMSO). Only 1 in 12 data points are shown to avoid overcrowded plot markers.

The catalytic activities of amyloids at pH 4 (without the addition of divalent ions) were studied in further detail because some amyloids composed of a peptide mixture (*e*.*g*. **12**/**13**, **12**/**14**, **13**/**14**, **28**/**29,** and **12**/**21**) were more active than both the positive control and **27** (Fig 1, condition 8). Their activity without zinc(II) and without the HXH motif, indicates a distinct mechanism. One problem to be resolved for a detailed analysis was that the protonation of the chromophore 4NPA at pH 4 precludes direct observation of the hydrolysis activity at 400 nm. In a first attempt, the identification of active amyloids was realized by indirect measurements, in which the reaction mixture is allowed to proceed at pH 4 for a given time before adjusting the mixture to pH 7 to measure a single time point. After 3 days of measurements it was clear that even the most active hits from the screen were ~500 times less active than the most active ones at pH 7.3, however with the background hydrolysis also reduced at pH 4 we identified a few amyloids composed of peptide mixtures that had more activity than the amyloids composed of their individual component peptides alone (Fig 1). We selected the peptide mixture **12**/**21** for further study with purified peptides. Based on the amount of soluble material remaining in the supernatant of a 100,000 g centrifugation, we found that at 40 μM, **12** began to aggregate within minutes at pH 4 while **21** remained soluble for several days. The 1:1 mixture of **12** and **21** aggregated more slowly than **12** alone with about 90% of the peptide in the 100,000 g pellet after 24 h. Furthermore, the activity of the peptide mixture only appeared after overnight incubation and, upon centrifugation, migrated with the 100,000 g pellet. Because of the low sensitivity of the photometric assay in combination with the error introduced by having to adjust the pH for each measurement, we utilized an NMR-based method to follow the kinetics of 4NPA hydrolysis at pH 4. Using this assay we could measure the reaction rate constant of the buffer *k* = 588.03 ± 0.39 × 10^−9^ s^-1^, the ratio of *k*_cat_ and *K*_M_ for the **12**/**21** amyloids (Table 4 and Fig 4) and, additionally, derive a turnover number of 7.5 for the peptide mixture amyloid (**12**/**21**). The results presented in Fig 4 and Table 4 show that the combination of peptides **12** and **21** has a higher *k*_cat_/*K*_M_ than either of the single peptide amyloids. The formation of a mixed amyloid can therefore be assumed considering the cooperative activity of the **12**/**21** mixture as well as faster aggregation of **21** in the presence of **12**. It is interesting to note that the activity of these amyloids is independent of zinc(II) and that neither **12** nor **21** contain the HXH motif thereby suggesting a reaction mechanism distinct from the one suggested by Rufo *et al*. for the most active amyloids (at pH 7.3) [23].

**Table 4:** Esterase activity of selected peptides at pH 4.3.

	Composition	Symbol^1^	*k*_cat_/*K*_M_ (10^-6^M^-1^s^-1^)
**21**	Ac-YV**S**V**S**V**H**V-CONH_2_	◇	1598±20
**12**	Ac-YV**H**V**H**V**D**V-CONH_2_	□	6145.6±8.5
**12**/**21**	Mixture	○	14987±12

^1^ Symbols used in Fig 4.

## Conclusions

Two types of strategies can be applied to search for novel peptide catalysts: design-based and library-based. While both strategies have their own advantages, they are not mutually exclusive, and any single approach can profit from both strategies. With this in mind we have developed an efficient way to screen a large array of amyloids for any activities that can be measured with a high-throughput detector. We have exploited the strengths of a library-based approach while retaining full control over the size and contents of the library. By working with crude peptides from SPPS and employing a solubilization condition applicable to all individuals in the library, we have produced a scalable procedure for libraries of amyloids by design. The amount of peptide required for each condition to be tested is on the order of 10 μg and so a typical small-scale peptide synthesis is sufficient for more than one hundred assay conditions. Because all of the necessary procedures from peptide production to fibrillization to activity assay are automatable and therefore amenable to HTS, our results pave the way to large screens of *de novo* functional amyloids.

The screens constructed here are based on a set of 94 peptides and binary peptide mixtures. In these screens the catalytic activity of several amyloids has been established. Since under a given condition usually several peptide amyloids showed activity, it appears that amyloid-based catalysis may be a rather common phenomenon, not requiring highly evolved sequences and/or specificity. Furthermore, despite the small sequence space tested it is notable that two different catalytic mechanisms were identified: one requiring the presence of zinc(II) (at pH 7.3) and one independent of zinc(II) (at pH 4). In addition, some of the amyloids display catalytic activity under extreme conditions such as high temperature, high salt, and high content of organic solvent. These polypotent properties make the amyloid an attractive platform for prebiotic enzymes in theories on mechanisms for the origin of life. The present involvement of a His residue for catalytic activity is, however, regarded as problematic in the quest for prebiotic enzymes since His is expected to have been a scarce prebiotic amino acid [34]. Thus, as a next step, amyloid libraries composed solely of prebiotically relevant amino acids will be constructed and the activities thereof will be screened following the strategies established here.

## Materials and Methods

### Peptide synthesis

The peptides were synthesized in an Apex 396 Parallel Synthesizer from AAPPTec with a 48-well reactor using standard FMOC solid phase chemistry on 40–50 mg rink-amide resin (0.88 μmol/mg) from Bachem. The resin was swelled in 100% DMF for 25 min and then deprotected using 20% piperidine in DMF. The coupling reaction comprising FMOC-AA, HCTU and DIEA (210 mM, 185 mM and 420 mM, respectively) in 37% DMF in NMP was repeated twice (20 min each) for each residue with a NMP wash in between. After coupling the resin was washed 3 times with DMF and then deprotected 5 times for 4 min with 20% piperidine. After deprotection, the resin was washed twice with DMF and then twice with NMP before the next coupling. After the final deprotection step, the peptides were acetylated with 10% Ac_2_O in DCM and DMF (1:1) and then washed 3 times with MeOH. After cleavage from the resin in 2 ml 90% TFA, 5% DCM, 2.5% TIS and 2.5% H_2_O for 2 h, the samples were precipitated with 45 ml cold diethyl ether, washed once with ether and then resuspended in 40 ml 50% CH_3_CN. The samples were frozen on liquid nitrogen and lyophilized. The identity and purity of the resulting polypeptides were evaluated by MALDI FT-ICR MS on a solariX 94 (Bruker) and reversed phase HPLC. Note that, unless otherwise noted, all experiments were conducted with fibrillized crude SPPS product.

### Peptide quantitation

For peptides that contain Tyr the concentration of the peptide was measured by diluting a 3–5 mM DMSO stock of the peptide 20-fold into 10 mM HCl and quickly measuring the UV spectrum to minimize the potential for aggregation (ε_276_ = 1500 M^-1^cm^-1^). Peptide **36** was quantitated during purification by C18 reversed phase HPLC using a calculated [35] extinction coefficient of ε_214_ = 13963 M^-1^cm^-1^. The quantification of the VS peptides in Table 1 and peptide **34** was performed by total hydrolysis in 6 M HCl at 160°C for 2 h. The hydrolyzed samples were analyzed by C18 reversed-phase and the amount of Val or Ile was calculated by comparing the chromatographic peak areas to amino acid samples of known quantity.

### Fibrillization

In order to accurately quantify the concentration of each peptide and to be able to initiate the fibrillization from a soluble peptide solution, generic solubilization conditions that would work with all of the peptides of the amyloid library were established. This was achieved for a peptide concentration of 4 mM in the two following solubilization conditions: (i) a solution of 99% DMSO with 1% TFA and (ii) a solution of 10 mM NaOH in 90% DMSO and 10% H_2_O. Peptides with a single Asp and two Ser residues were, however, only soluble in the second solution. By using a universal solubilization condition, the peptide mixtures could be prepared in a soluble state using that same condition.

Unless otherwise noted, fibrillization was initiated by diluting the solubilized peptides (and peptide mixtures) 25 times into the fibrillization condition yielding a peptide concentration of 160 μM. The various fibrillization conditions used are mentioned in the main text and in Table B in S1 File.

### Circular dichroism (CD)

CD measurements were carried out on a Jasco J815. Samples were prepared by centrifuging the amyloid library at 25,000 g for 10 min and then removing the supernatant. The pellets were resuspended at an approximate concentration of 100 μg/ml in 10 mM HEPES pH 7.3 (for mixtures from 50 mM HEPES) or 10 mM NaOAc pH 4 (for mixtures from 50 mM NaOAc), the suspensions were then treated with an ultrasonic probe for 5 s, upon which the solutions were placed in 1 mm cuvettes, diluted as necessary to maintain the detector voltage below 600 V and scanned from 260–190 nm at 50 nm/min, 1 nm band-pass, 2 second integration, averaged over 3 repetitions.

### Transmission electron microscopy (TEM)

The samples of the amyloid library (50 mM HEPES pH 7.3 or 50 mM NaOAc pH 4) were applied directly to glow-discharged carbon-coated grids, washed and then stained with uranyl acetate. Images were recorded on a Philips CM 12 electron microscope.

### Fourier transform infrared spectroscopy (FTIR)

Samples were prepared as for the CD measurements, but instead of re-suspending the samples after centrifugation, 3 μl of H_2_O were added to the pellet so that it could be placed onto the diamond ATR cell of a Bruker Alpha-p ATR-FTIR spectrometer followed by air-drying before measuring the spectra. Blank subtraction was performed with an empty cell (air) and all measurements were performed without the pressure applicator.

### Thioflavin T fluorescence (ThT)

The samples of the amyloid library (50 mM HEPES pH 7.3 or 50 mM NaOAc pH 4) were mixed 10:1 with 50 μM ThT and the increase in fluorescence was measured in a 384-well plate in a BMG Pherastar platereader (*ex*: 440 nm *em*: 480 nm). The background signal from the peptides without ThT was subtracted from the one with the ThT present in the sample. Due to the large dynamic range of the signals among the many samples, the measurements were made at two different gain settings to ensure that all of the signals fell within the measureable range.

### Fiber diffraction

The samples were centrifuged, the pellet resuspended (ultrapure water or 10 mM NaOAc pH 4) and centrifuged again. The washed pellets were resuspended in a minimal amount of water and 4 μl of the suspension were pipetted between the melted ends of two glass capillaries, which were then left to dry overnight in a sealed petri dish. The dried material was placed in an X-ray beam (Rigaku MicroMax-007HF) at room temperature for a 20 min exposure and diffraction data was collected on a Mar345 image plate detector.

### Plate reader 4NPA hydrolysis assay

The reaction buffer consisted of the same buffer as the fibrillization buffer without ZnCl_2_ and with 1.33 mM 4NPA prepared from a 100 mM stock in CH_3_CN. The samples of the amyloid library were mixed 1:3 with the reaction buffer yielding 40 μM peptide and, when present 250 μM zinc(II). The absorbance measurements were performed in a BMG Pherastar platereader with a 400 nm band-pass filter (10 nm bandwidth) in 96-well Nunclon Delta surface plates or 384-well Greiner Bio-One non-bind black μclear plates.

The kinetics assays performed on the purified peptides were prepared similarly except that the peptide concentration was 20 μM in the reaction. Furthermore, the various substrate concentrations and replicate samples were randomly distributed over the plate to minimize systematic errors. Various 4NPA concentrations (*i*.*e*. 0.1 mM, 0.2 mM, 0.4 mM, 0.6 mM, 1.0 mM) were added from a 100 times stock so that the final CH_3_CN concentration was always 1%. The linear part of the hydrolysis activity was fit with an extinction coefficient of *ε* = 9700 M^-1^cm^-1^ and corrected for the background reaction rate of buffer alone (*v*_bg_ = 5.0 × 10^-6^ Ms^-1^) to obtain the initial reaction rate *v*_0_. The average of the initial reaction rates at each substrate concentration were then fit to the Michaelis-Menten type kinetics to obtain *k*_cat_, *K*_M_ and *k*_cat_/*K*_M_ always calculated per monomer peptide. The background reaction rate was obtained similarly using 5 replicates.

### NMR-based 4NPA hydrolysis assay

Purified peptides were solubilized in deuterated DMSO with 1% TFA at a concentration of 4 mM and subsequently diluted to 160 μM in 50 mM deuterated NaOAc buffer at pH 4.3. In the case of the peptide mixture fibrillization (**12**/**21**), each peptide was 80 μM. Fibrillization occurred over 5 days after which the sample was diluted to 40 μM total peptide in 50 mM buffer with 0.9 mM 4NPA (from a 90 mM stock solution in d_6_-DMSO) and a final concentration of d_6_-DMSO of 2%. From this reaction mixture 500 μl was transferred to a 5 mm NMR tube and measurements were performed in a 600 MHz Bruker NMR spectrometer equipped with a cryo-probe.

The hydrolysis kinetics were monitored in a continuous series of 1D-^1^H experiments each consisting of 32 scans at 25°C and each requiring 17 min. In this manner, several time segments lasting between 8 and 17 hours were collected over a period of 15 days. Between measurements, the samples were kept at 25°C.

Data processing and peak integration was performed with Topspin3.2 (Bruker). Relevant peaks used for the analysis were the 4 aromatic protons of both 4NPA and of its product (*p*-nitrophenol) as well as the methyl groups of 4NPA and acetic acid. The ratio of the sum of integrals for the product protons to the sum of integrals for all protons was used to calculate the substrate concentration. The time-dependence of the substrate concentration was fit to a first order kinetic derived from a Michaelis-Menten type kinetics in a regime for which the substrate concentration is lower than the Michaelis-Menten-constant *K*_M_.

## Supporting Information

S1 File Supplemental file. (PDF)
