# Supplementary material for: Towards Prebiotic Catalytic Amyloids Using High Throughput Screening
Source: PLoS One. 2015 Dec 9;10(12):e0143948. doi: 10.1371/journal.pone.0143948 (PMC4674085; doi:10.1371/journal.pone.0143948)
Supplement: S1 File — (PDF) [file pone.0143948.s001.pdf]

Table A and associated data: Biophysical characterization of library peptides.

	Sequence	pH 7.3 ThT[1]	CD[2]	FTIR[3]	EM[4]	pH 4.0 ThT	CD	FTIR	EM	X-ray[5]
**1**	Ac-YV**D**V**D**V**D**V-CONH$_2$	1	–	–		89	β^{++}	1625	+	
**2**	Ac-YV**S**V**D**V**D**V-CONH$_2$	24	–	1624		322	β^{++}	1624	+	cross-β
**3**	Ac-YV**D**V**S**V**D**V-CONH$_2$	2	–	–		120	β^{++}	1623	+	
**4**	Ac-YV**D**V**D**V**S**V-CONH$_2$	3	–	–		160	β^{++}	1618	+	
**5**	Ac-YV**S**V**S**V**D**V-CONH$_2$	322	β^{+}	1619		322	β^{++}	1618	+	
**6**	Ac-YV**S**V**D**V**S**V-CONH$_2$	309	β^{+}	1618		322	β^{++}	1622	+	
**7**	Ac-YV**D**V**S**V**S**V-CONH$_2$	208	–	–		128	β^{++}	1619	+	
**8**	Ac-YV**H**V**H**V**H**V-CONH$_2$	126	β^{+}	1627	+	1	–	–	–	cross-β
**9**	Ac-YV**S**V**H**V**H**V-CONH$_2$	82	β^{++}	1624	+	1	–	–		
**10**	Ac-YV**H**V**S**V**H**V-CONH$_2$	29	β^{+}	1623	+	1	–	–		
**11**	Ac-YV**H**V**H**V**S**V-CONH$_2$	18	β^{++}	1624	+	1	–	–		
**12**	Ac-YV**S**V**S**V**H**V-CONH$_2$	183	β^{++}	1623		5	?	1621	+	cross-β
**13**	Ac-YV**S**V**H**V**S**V-CONH$_2$	0	β^{+}	1622	+	11	β^{++}	1621		
**14**	Ac-YV**H**V**S**V**S**V-CONH$_2$	322	β^{+}	1624	+	2	β^{+}	1623		cross-β
**15**	Ac-YV**D**V**H**V**S**V-CONH$_2$	114	β^{+}	1623	+	13	β^{+}	1622	+	
**16**	Ac-YV**D**V**S**V**H**V-CONH$_2$	177	β^{++}	1618	+	7	β^{++}	1619	+	
**17**	Ac-YV**H**V**D**V**S**V-CONH$_2$	163	β^{+}	1622	+	5	β^{++}	1622		
**18**	Ac-YV**S**V**D**V**H**V-CONH$_2$	31	β^{++}	1622	+	1	β^{++}	1622		
**19**	Ac-YV**S**V**H**V**D**V-CONH$_2$	142	β^{++}	1624	+	9	β^{++}	1624		
**20**	Ac-YV**H**V**S**V**D**V-CONH$_2$	98	β^{+}	–	+	23	β^{++}	1622	+	
**21**	Ac-YV**H**V**H**V**D**V-CONH$_2$	133	β^{+}	1626	+	1	?	–	+	
**22**	Ac-YV**H**V**D**V**H**V-CONH$_2$	44	β^{+}	1624	+	1	?	–	+	
**23**	Ac-YV**D**V**H**V**H**V-CONH$_2$	21	β^{+}	1624	+	1	–	1626	+	
**24**	Ac-YV**D**V**D**V**H**V-CONH$_2$	2	–	–	+	1	β^{++}	1626	+	
**25**	Ac-YV**D**V**H**V**D**V-CONH$_2$	2	–	–		9	β^{++}	1623	+	
**26**	Ac-YV**H**V**D**V**D**V-CONH$_2$	5	–	–		5	β^{++}	1624	+	
**27**	Ac-YV**A**V**H**V**H**V-CONH$_2$	129	β^{+}	1625	+	1	–	–		cross-β
**28**	Ac-YV**H**V**A**V**H**V-CONH$_2$	114	β^{+}	1624	+	1	–	–		
**29**	Ac-YV**D**V**H**V**A**V-CONH$_2$	183	β^{+}	1622	+	25	?	1618		
**30**	Ac-YV**A**V**D**V**H**V-CONH$_2$	42	–	1623	+	10	β^{++}	1624		
**31**	Ac-YV**H**V**D**V**A**V-CONH$_2$	259	β^{+}	1622		32	β^{++}	1621	+	
**32**	Ac-YV**H**V**A**V**D**V-CONH$_2$	322	β^{+}	1621		111	β^{++}	1621	+	
**33**	Ac-YV**A**V**H**V**D**V-CONH$_2$	67	β^{+}	1624		2	β^{++}	1623	+	

1. Fold increase in ThT fluorescence over background.
2. CD spectra classified as follows: (β^{+}) has minimum near 217 nm, (β^{++}) has minimum near 217 nm and a maximum near 197 nm, (–) signal too weak to interpret, (?) spectra not typical of any secondary structure.
3. The peak position in amide I region is given in cm^{-1}. (–) indicates that no peak was observed within the typical range of stretching frequencies for β-structure (between dashed lines at 1638 cm^{-1} and 1615 cm^{-1})[1].
4. (+) fibrils present or (–) absent in EM micrographs.
5. Diffraction images recorded for the aligned fibrils of 5 different peptides (see materials and methods).

Ac–YV**D**V**D**V**D**V–$CONH_2$ (**1**)

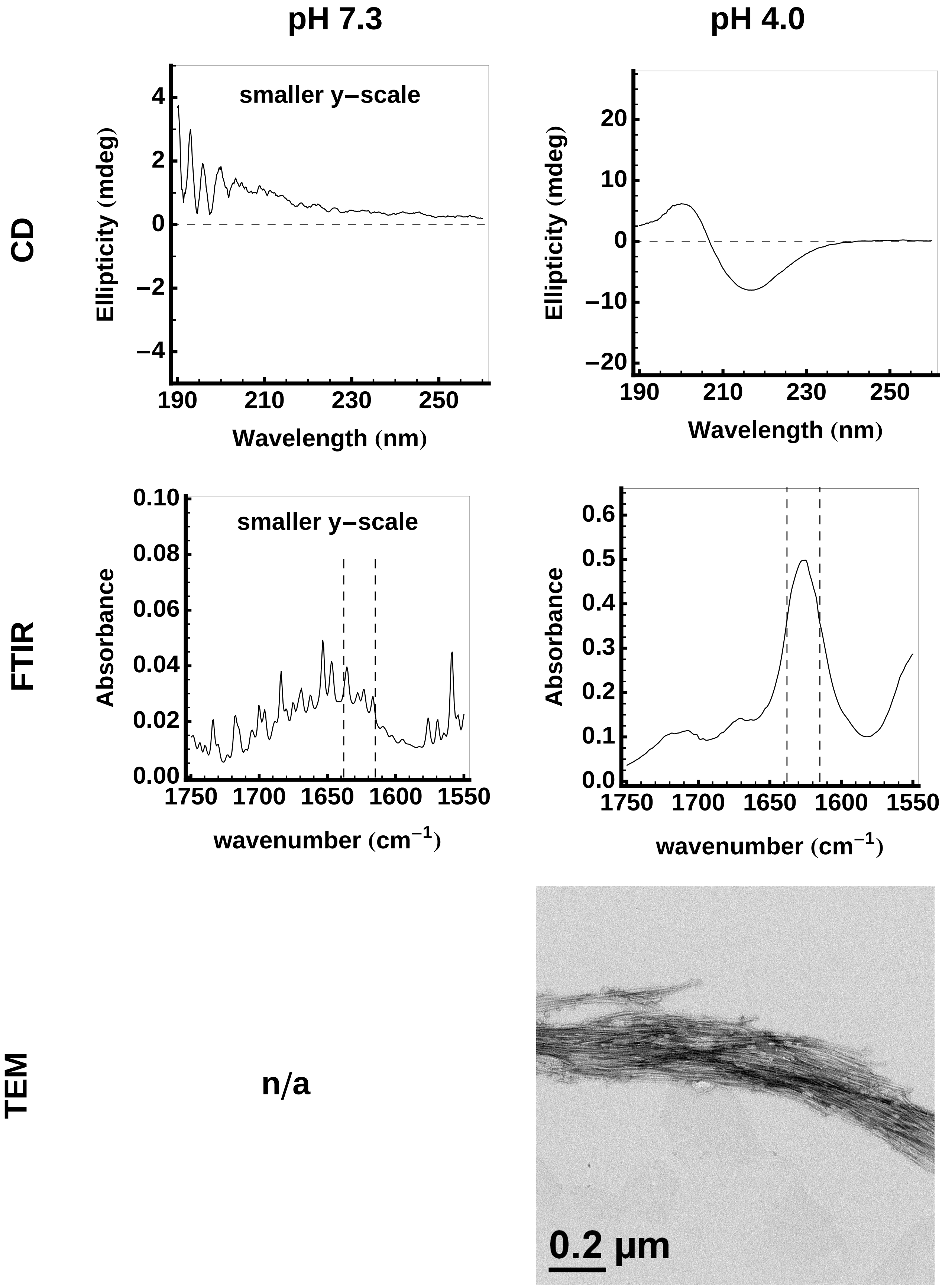

Ac−YV**S**V**D**V**D**V−CONH$_2$ (**2**)

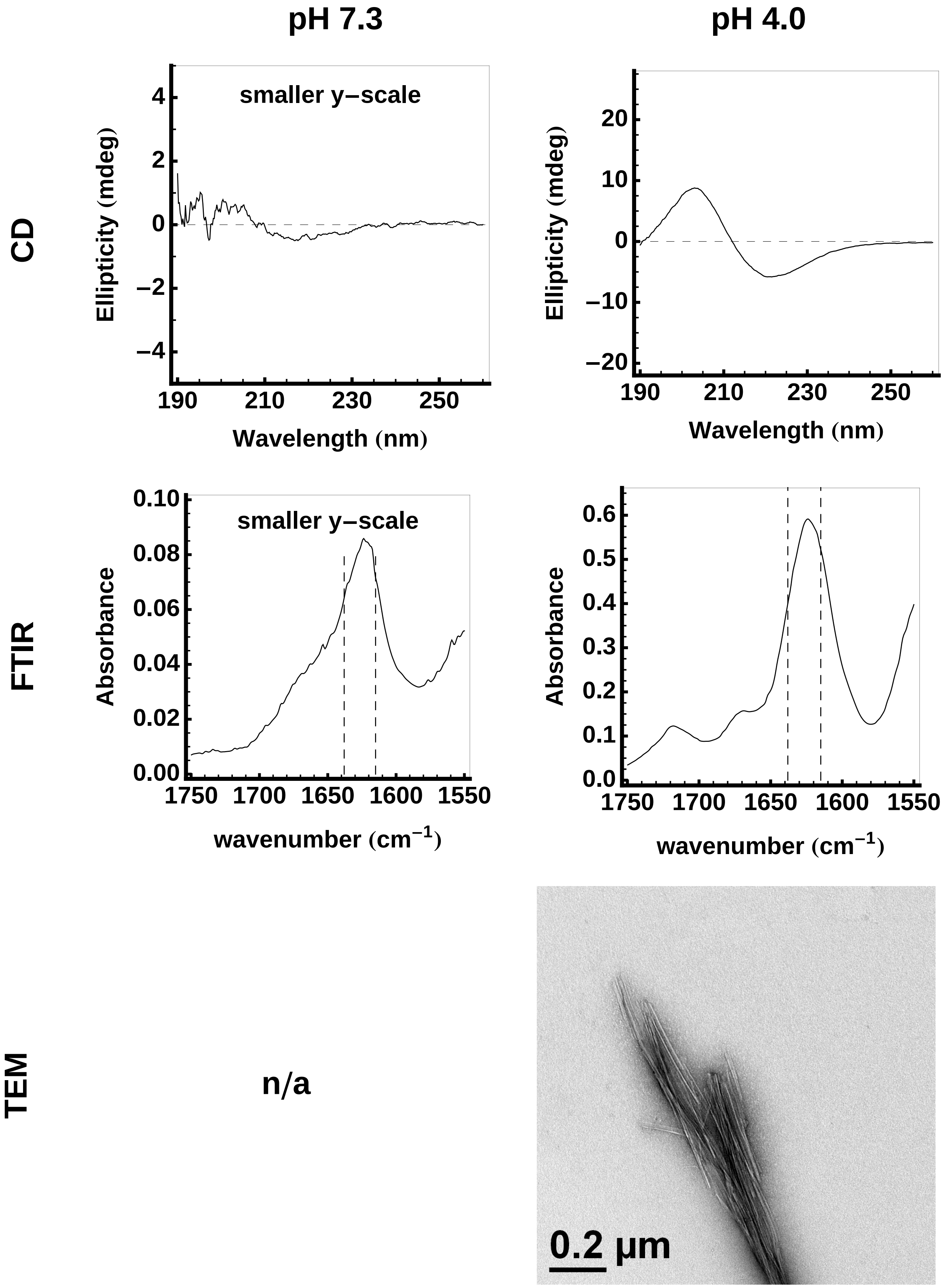

Ac–YV**D**V**S**V**D**V–CONH$_2$ (**3**)

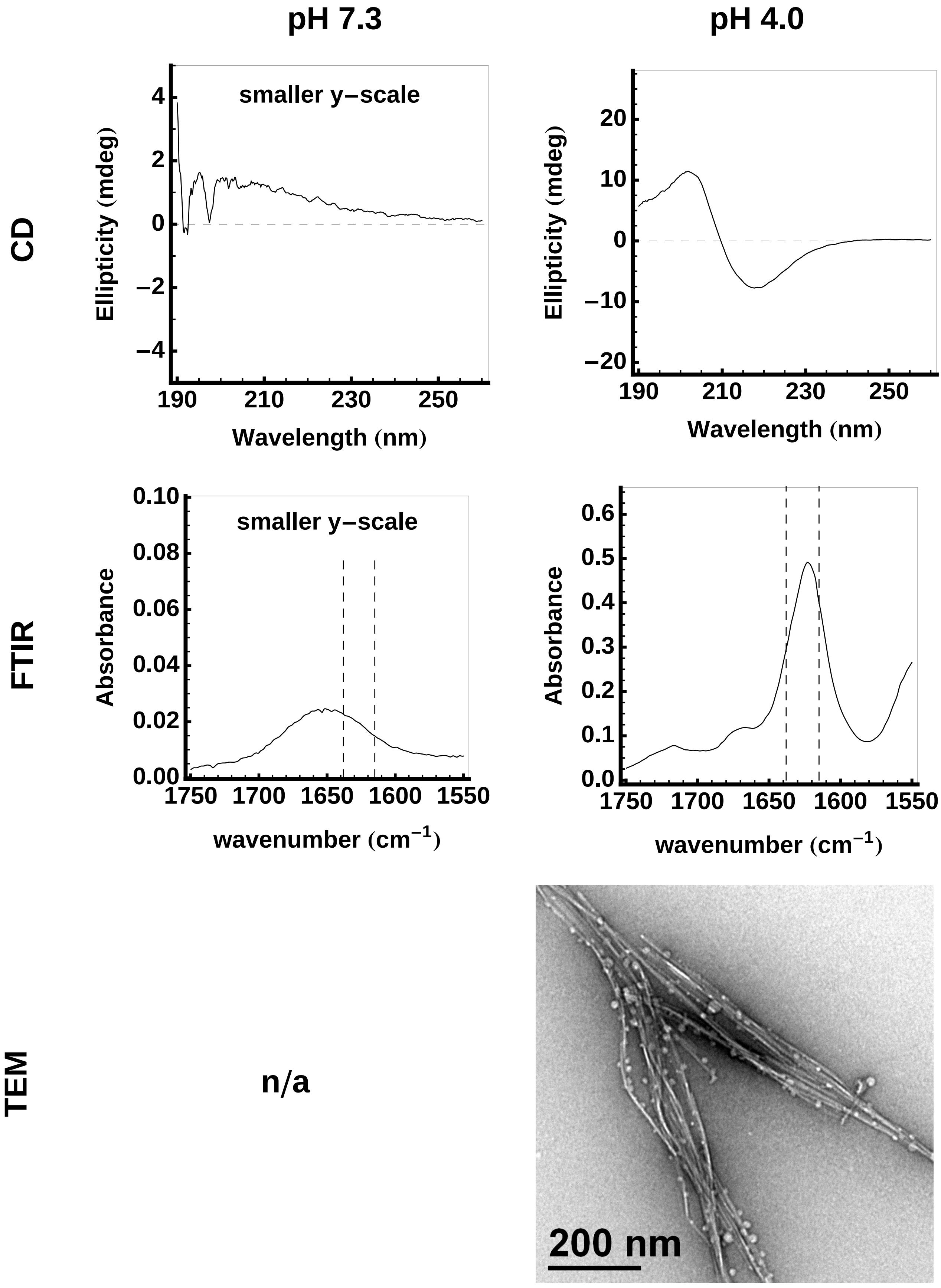

Ac–YV**D**V**D**V**S**V–CONH$_2$ (**4**)

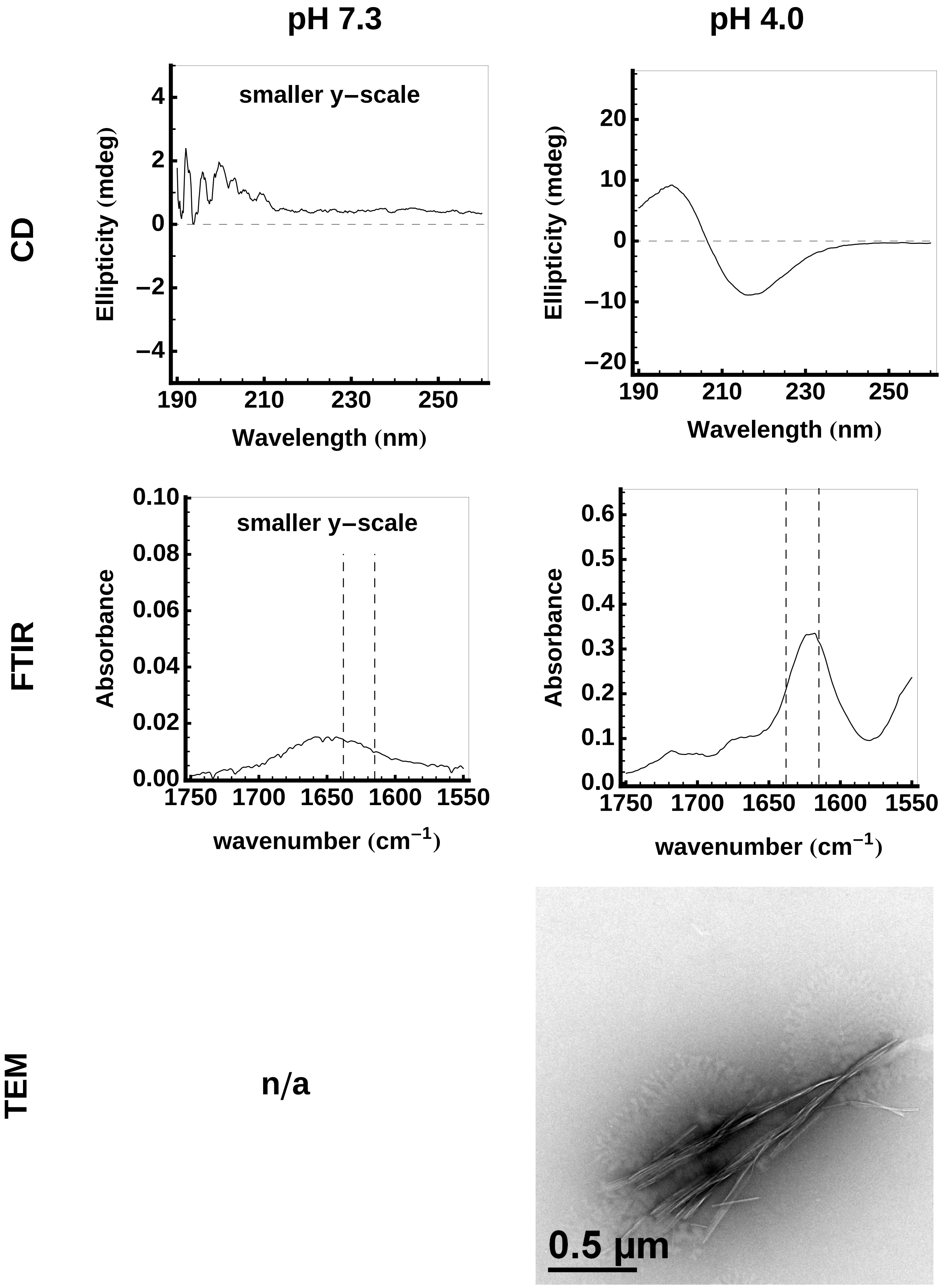

Ac−YV**S**V**S**V**D**V−$CONH_2$ (**5**)

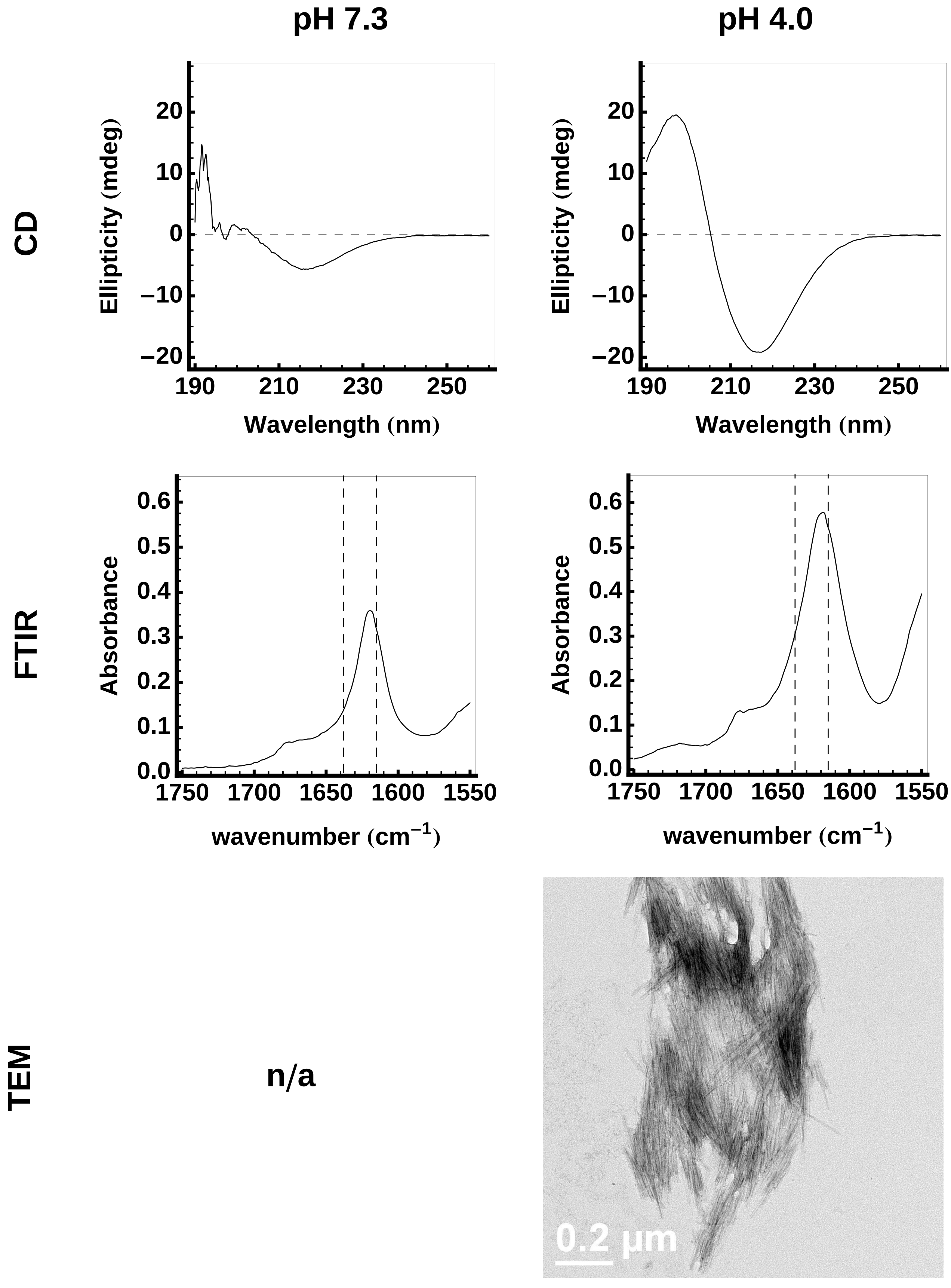

Ac−YV**S**V**D**V**S**V−$CONH_2$ (**6**)

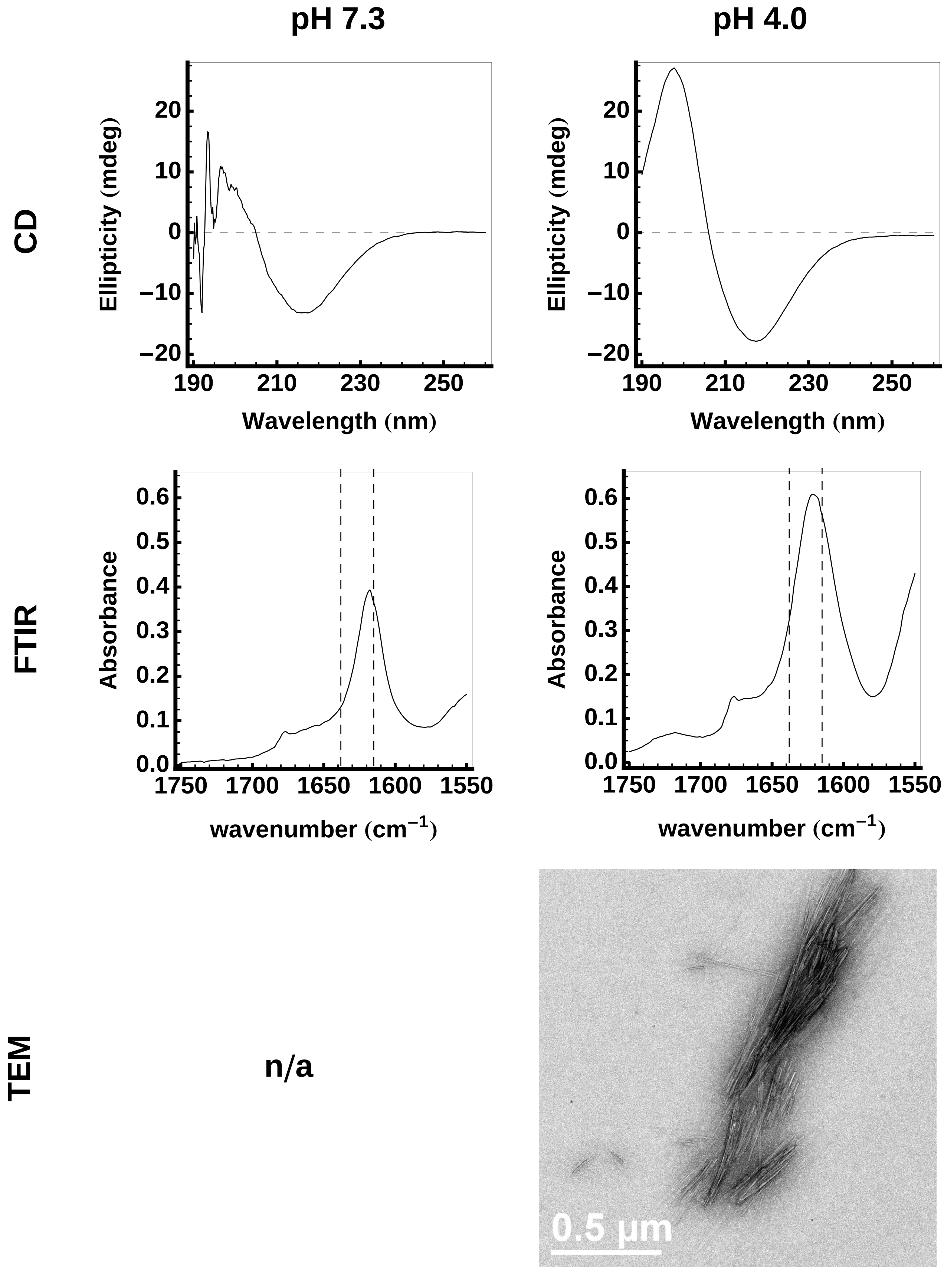

Ac-YV**D**V**S**V**S**V-$CONH_2$ (**7**)

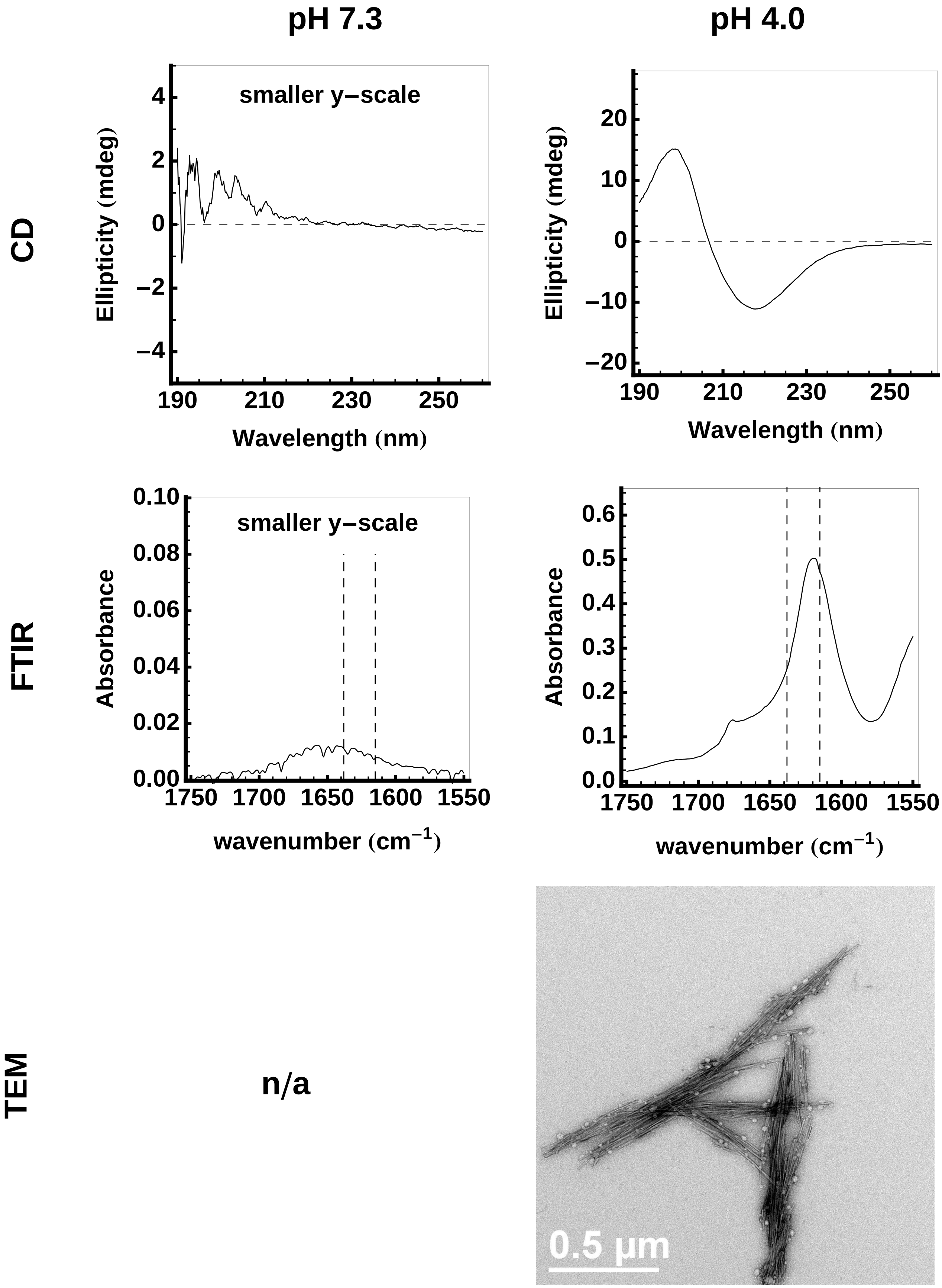

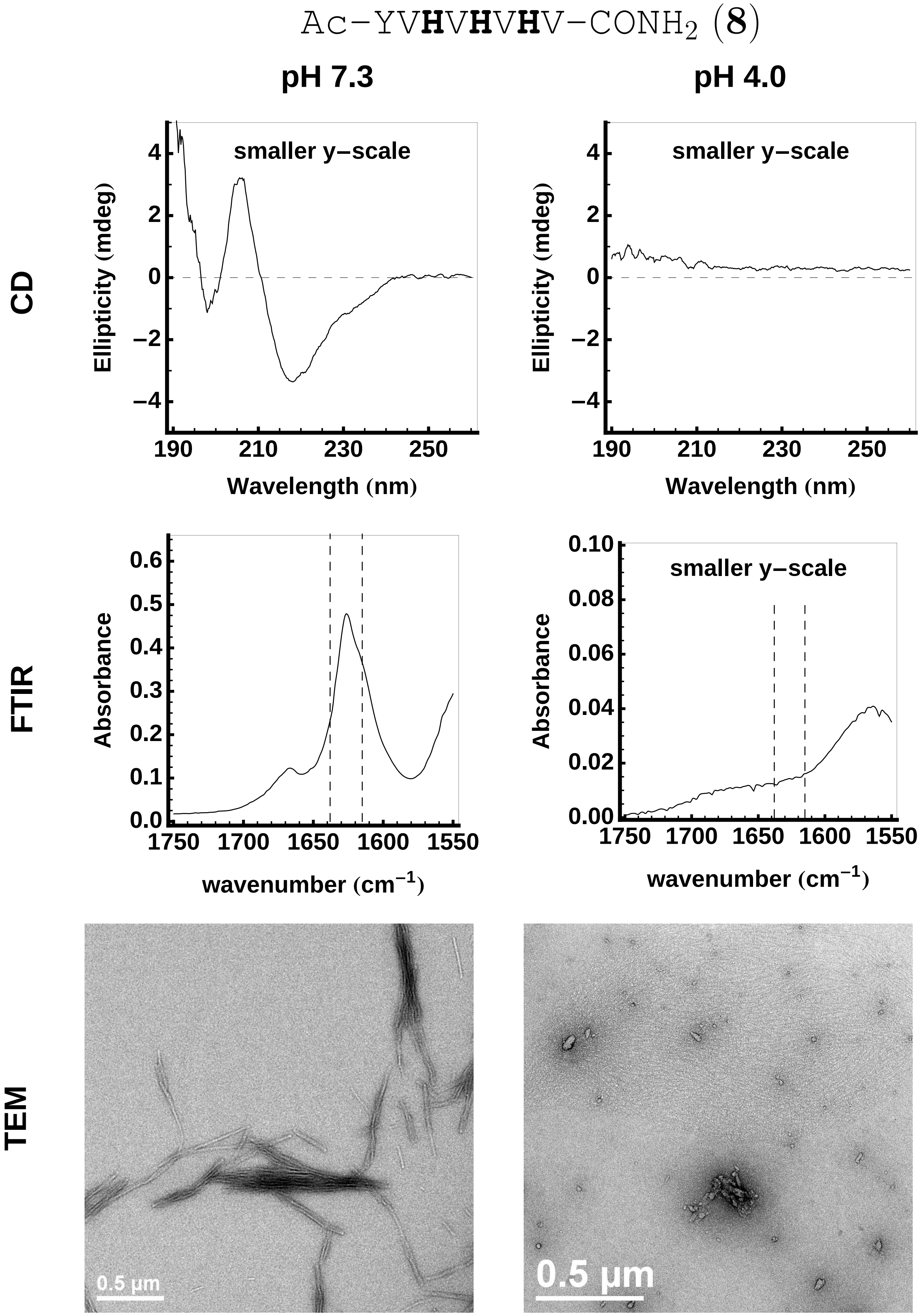
Ac–YVHVHVHV–CONH2 (8)
pH 7.3
pH 4.0
CD
FTIR
TEM
smaller y–scale
Ellipticity (mdeg)
Wavelength (nm)
Absorbance
wavenumber (cm−1)
0.5 µm

Ac−YV**S**V**H**V**H**V−CONH$_2$ (**9**)

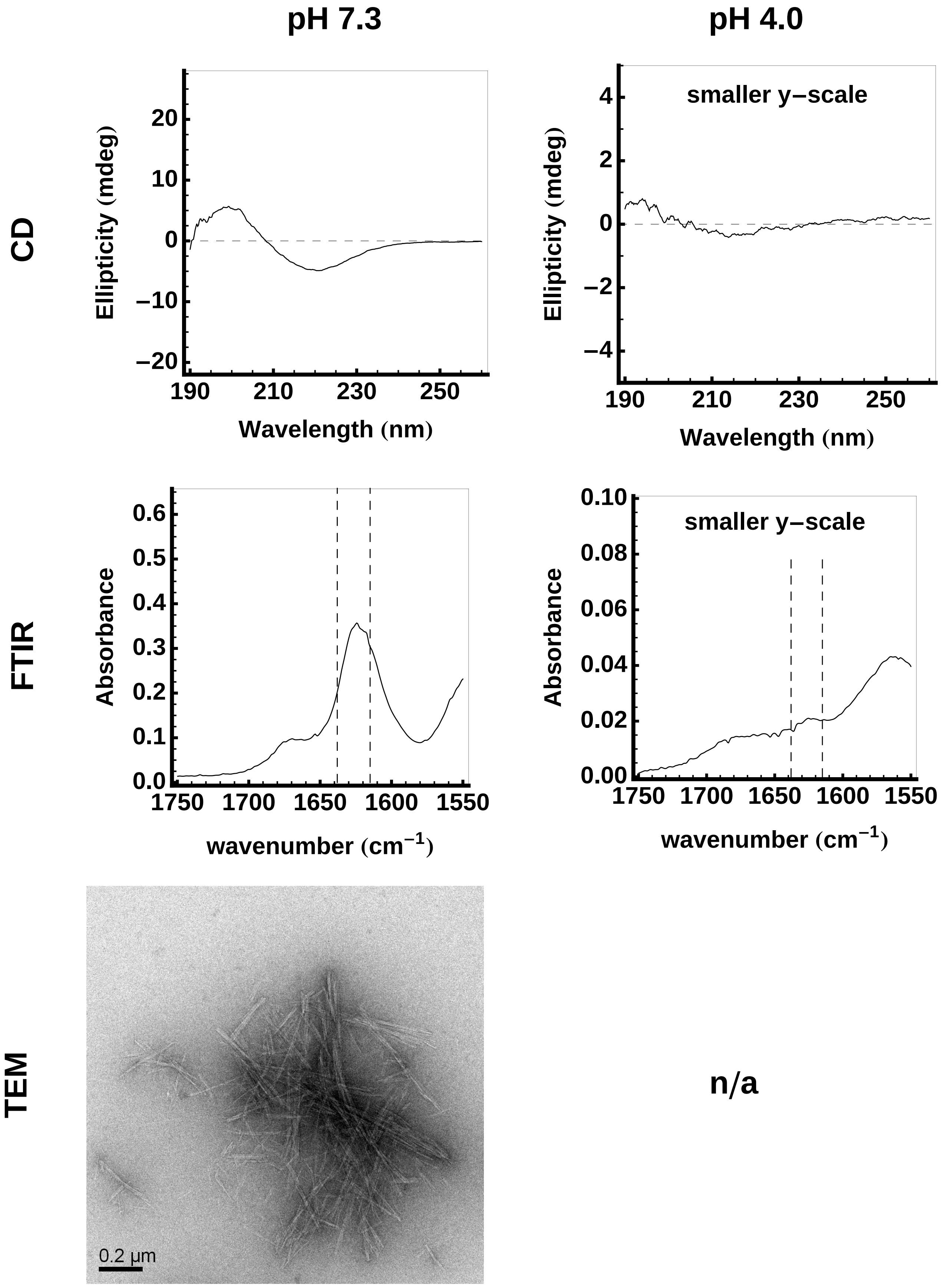

Ac−YV**H**V**S**V**H**V−$CONH_2$ (**10**)

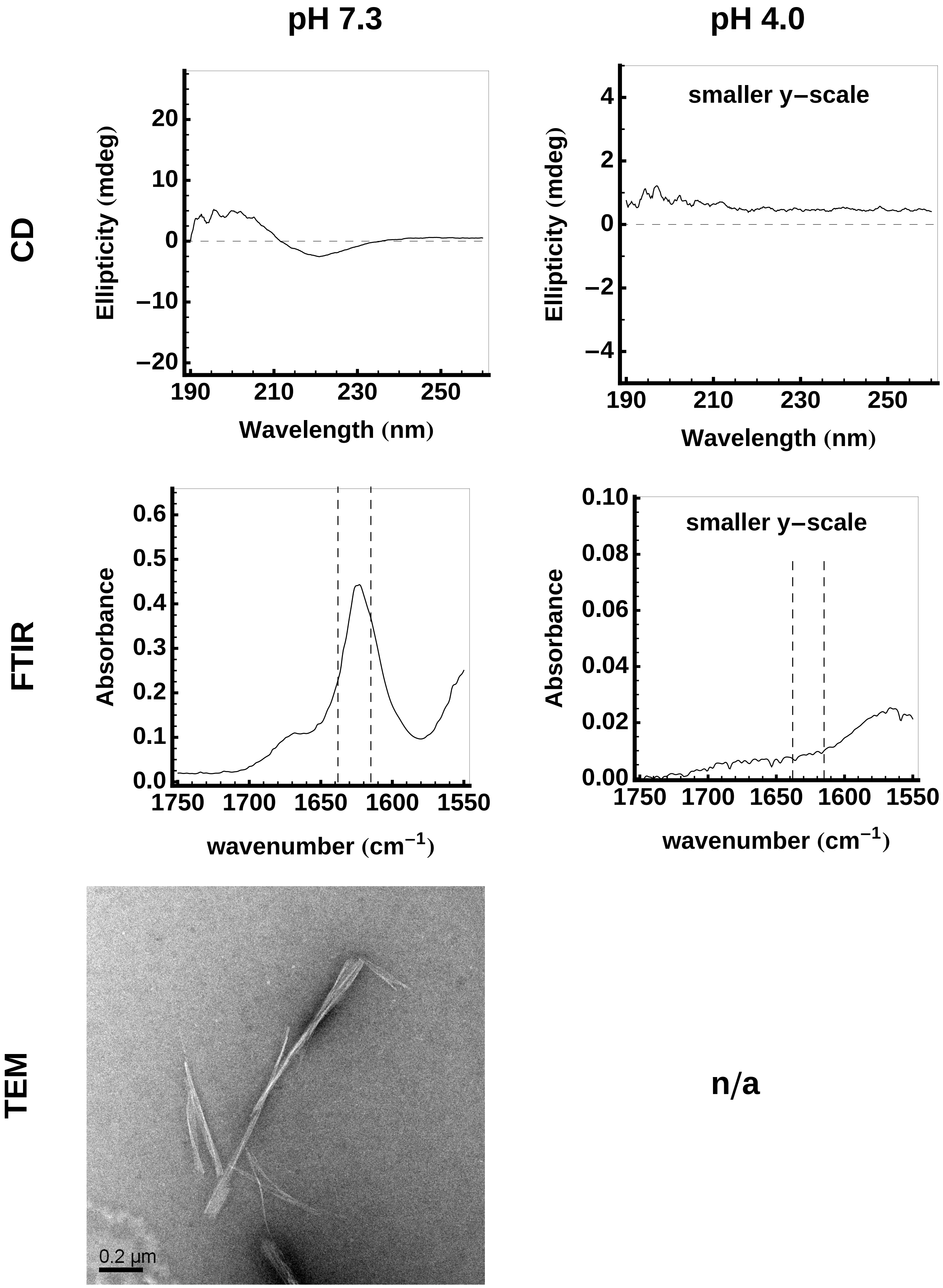

Ac–YV**H**V**H**V**S**V–CONH$_2$ (**11**)

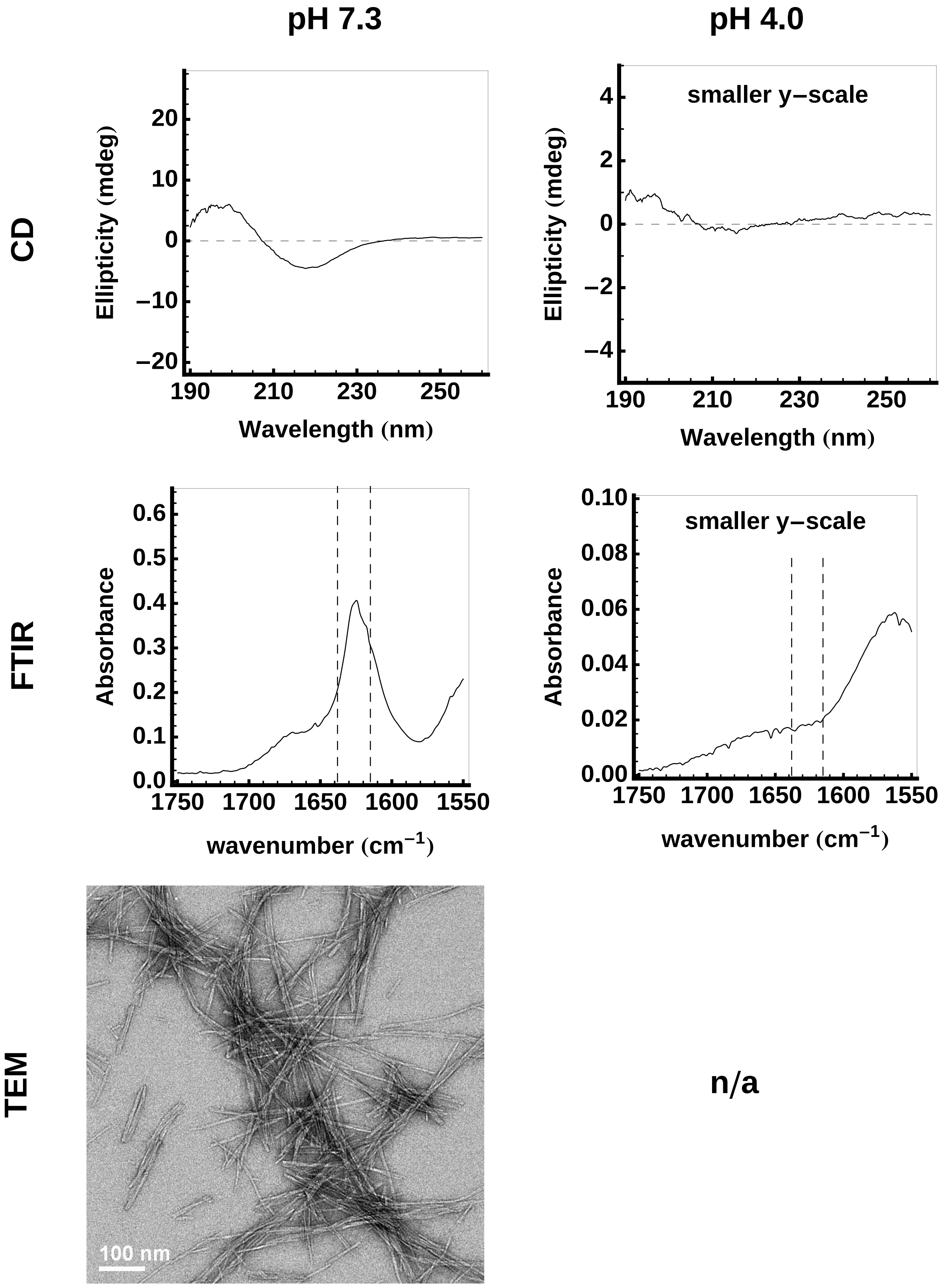

Ac-YV**S**V**S**V**H**V-CONH$_2$ (**12**)

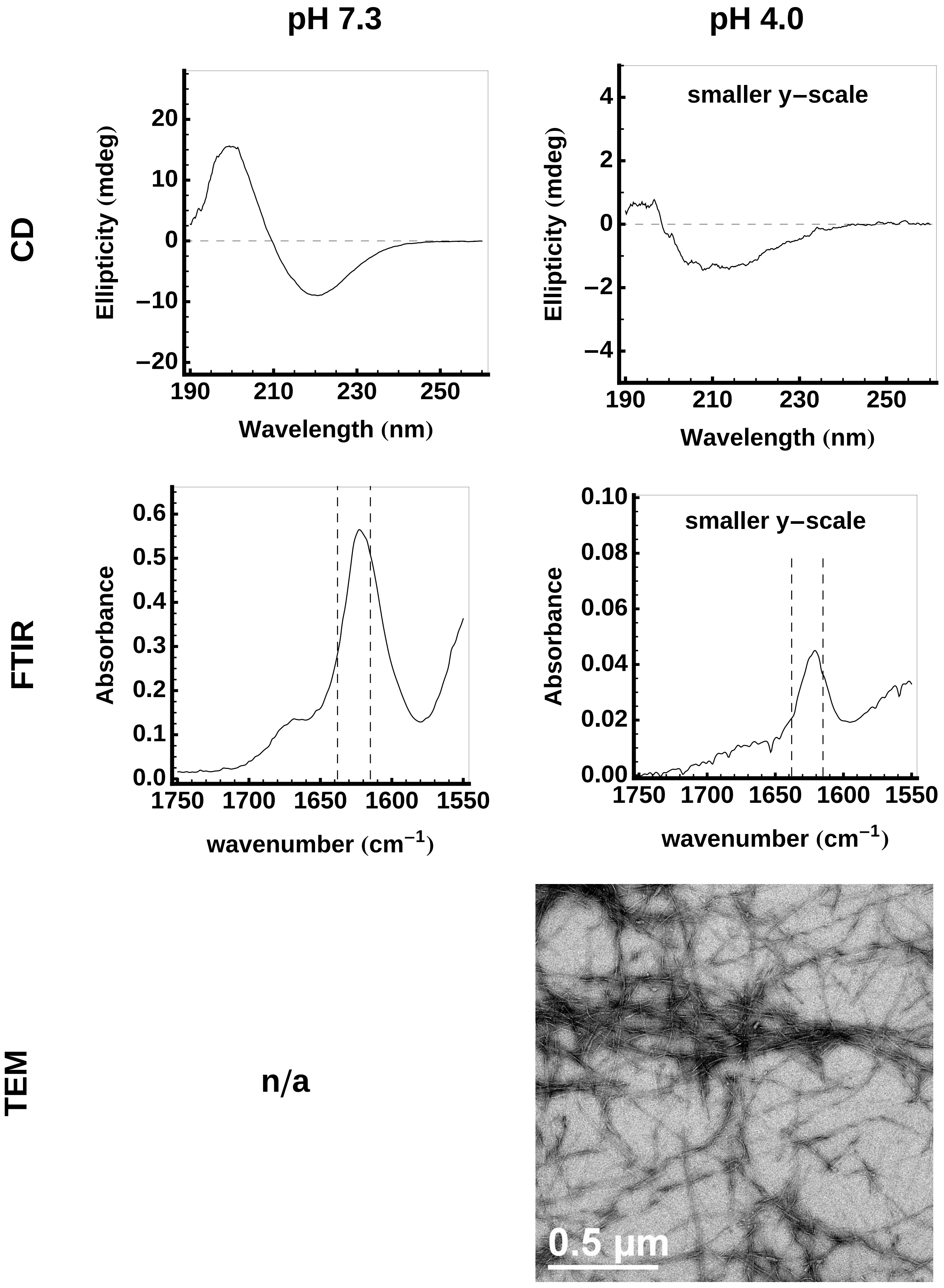

Ac−YV**S**V**H**V**S**V−$CONH_2$ (**13**)

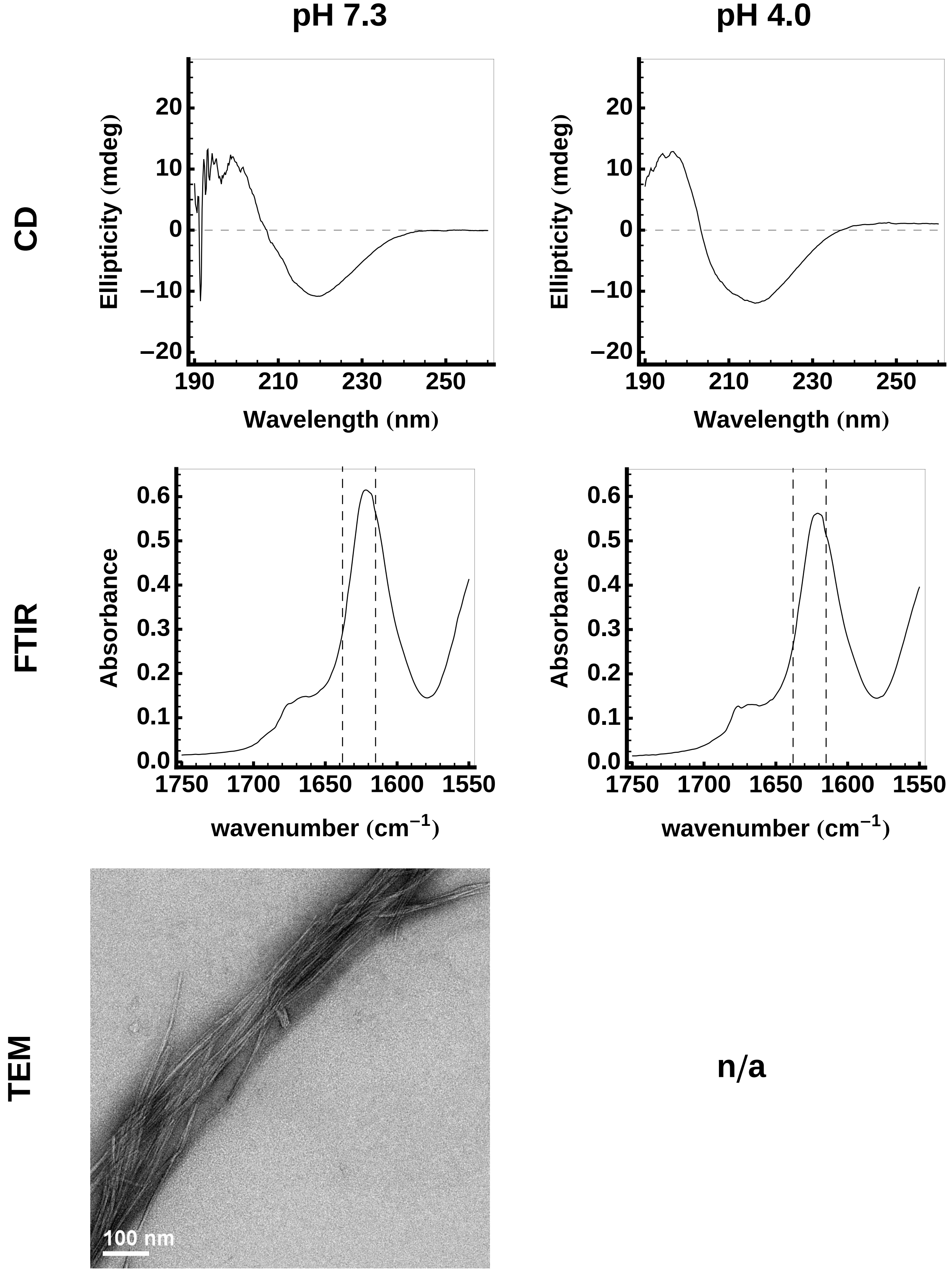

Ac–YV**H**V**S**V**S**V–CONH$_2$ (**14**)

	**pH 7.3**	**pH 4.0**
**CD**	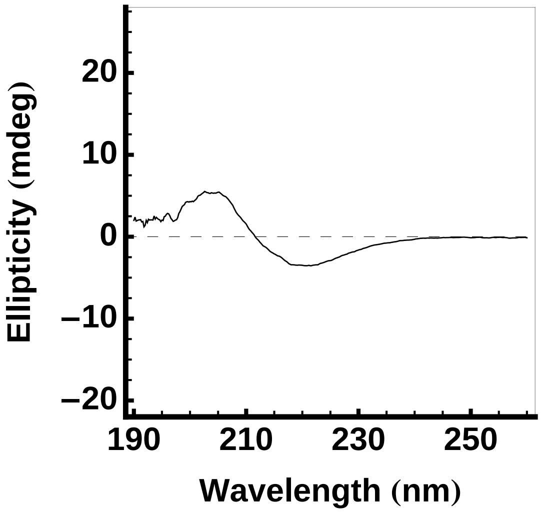	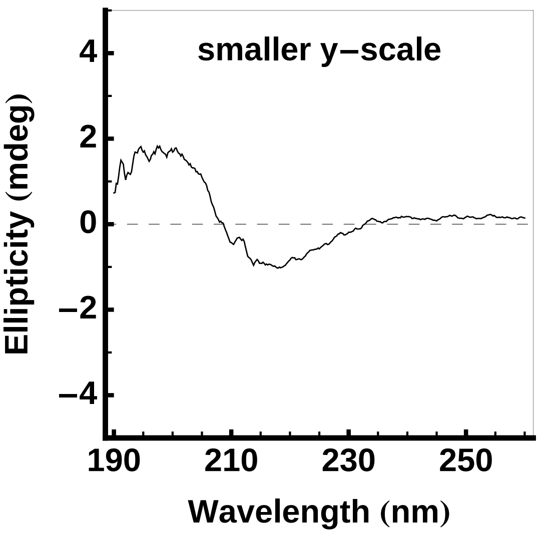
**FTIR**	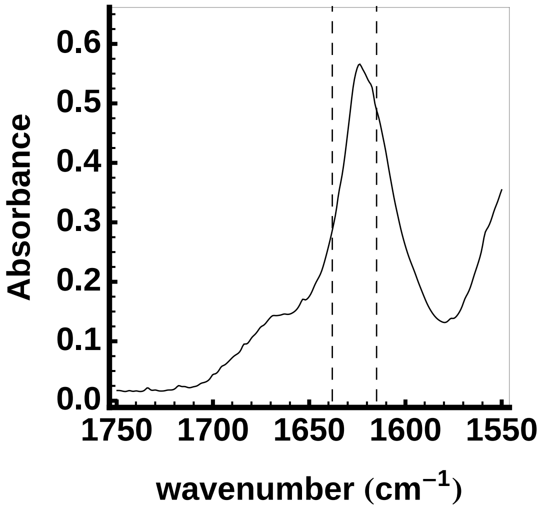	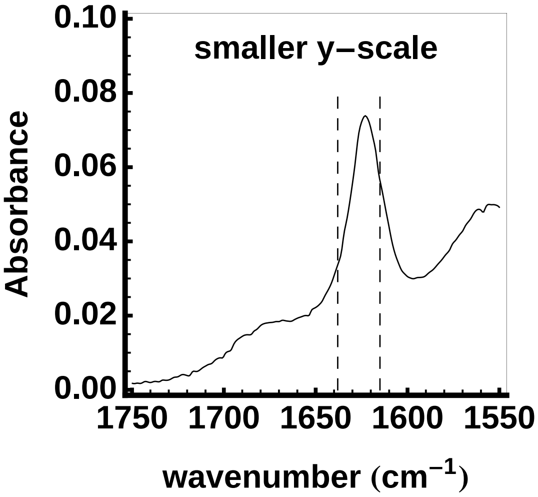
**TEM**	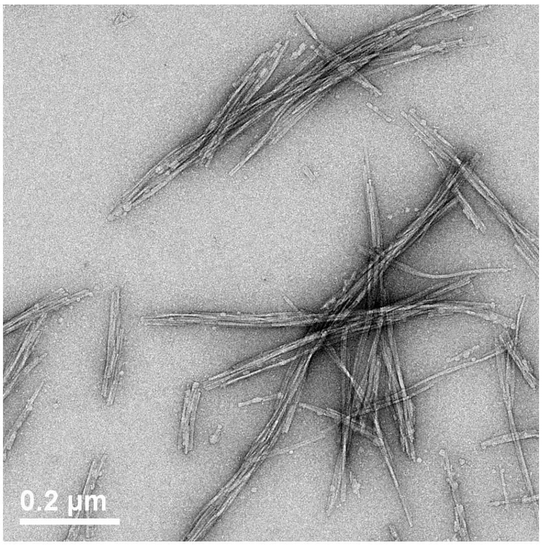	**n/a**

Ac−YV**D**V**H**V**S**V−CONH$_2$ (**15**)

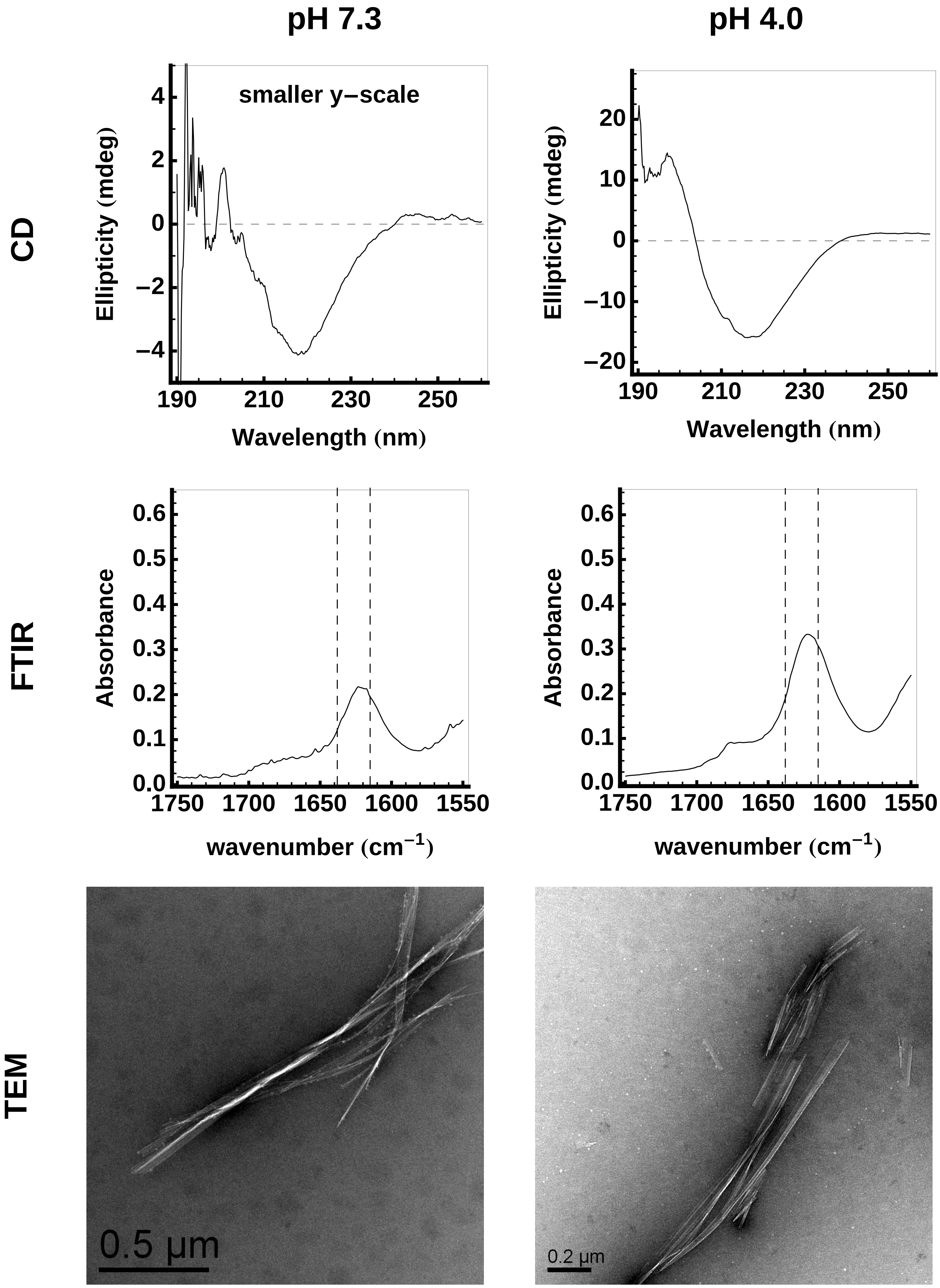

Ac−YV**D**V**S**V**H**V−CONH$_2$ (**16**)

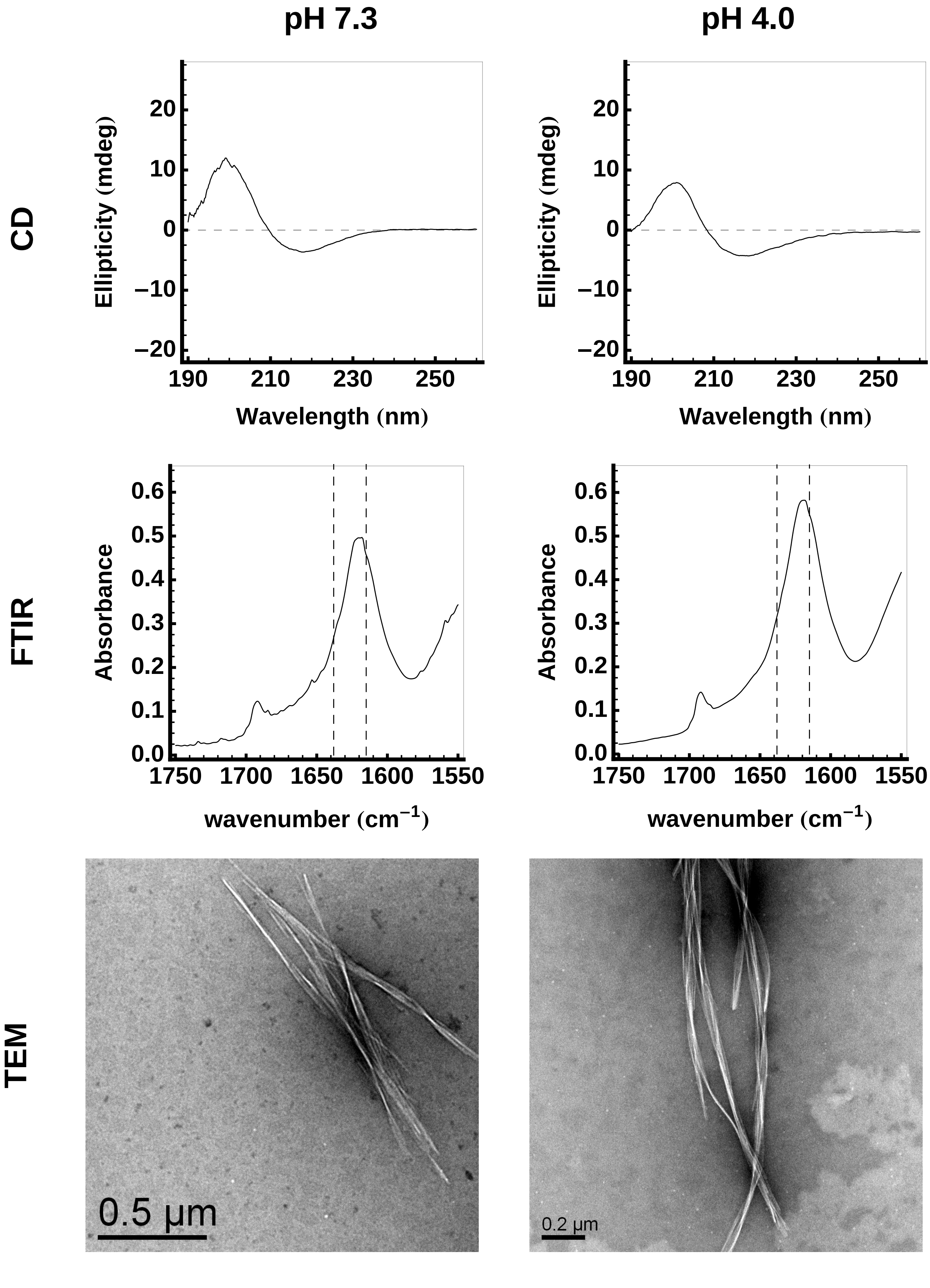

Ac–YV**H**V**D**V**S**V–$CONH_2$ (**17**)

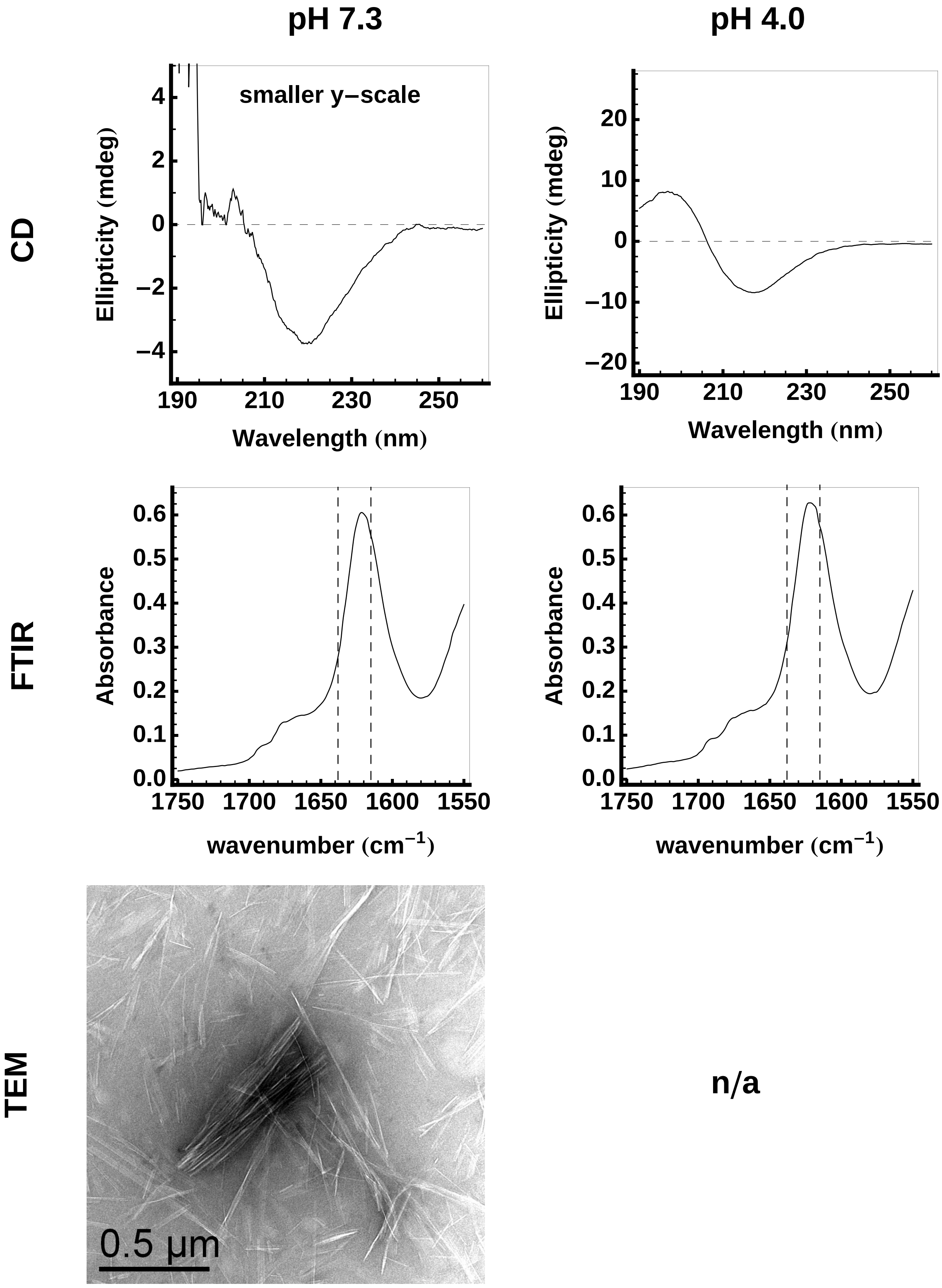

Ac-YV**S**V**D**V**H**V-CONH$_2$ (**18**)

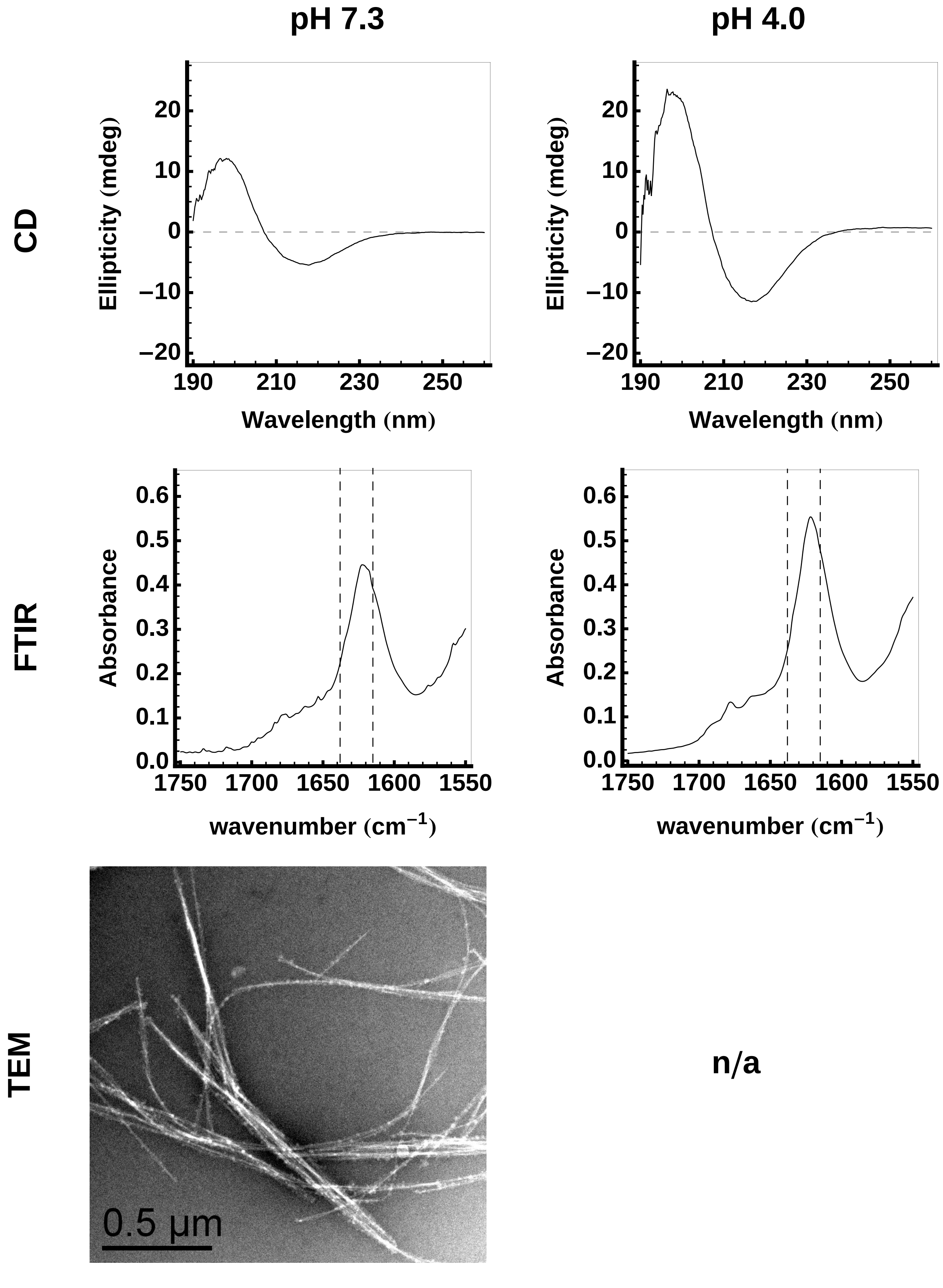

Ac–YV**S**V**H**V**D**V–$CONH_2$ (**19**)

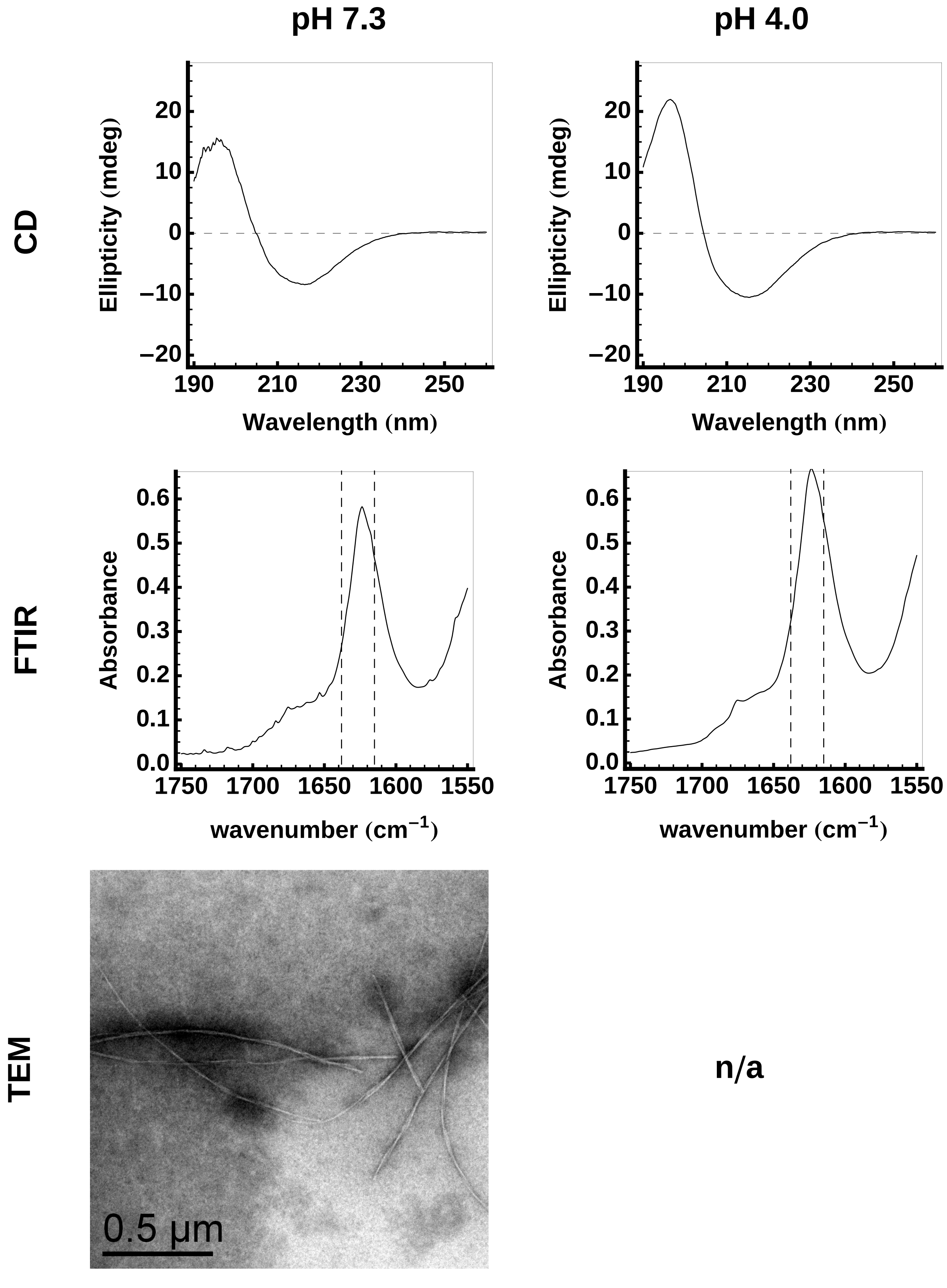

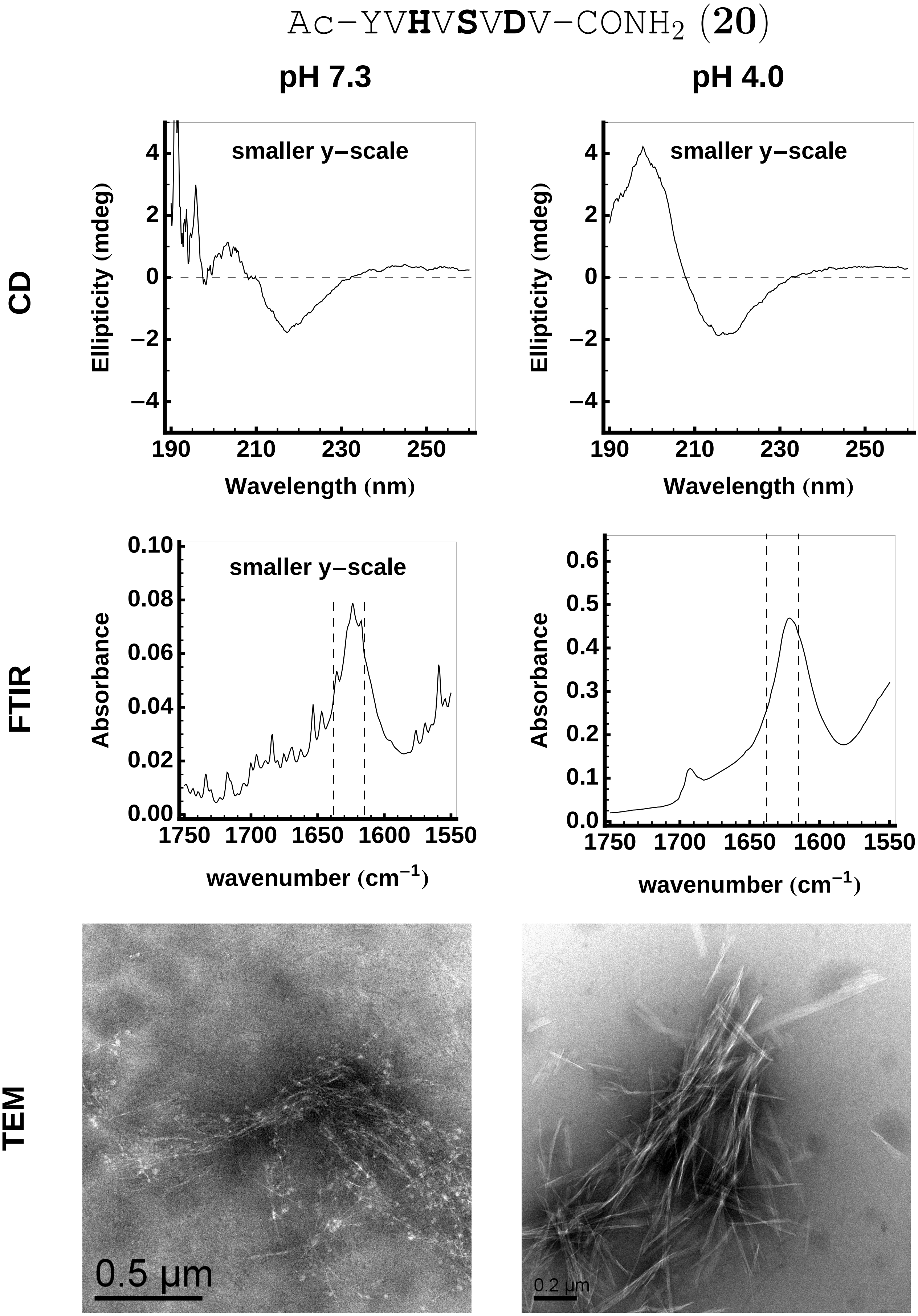

Ac−YVHVSVDV−CONH2 (20)
pH 7.3
pH 4.0
CD
FTIR
TEM
smaller y−scale
Ellipticity (mdeg)
Wavelength (nm)
Absorbance
wavenumber (cm−1)
0.5 μm
0.2 μm

Ac−YV**H**V**H**V**D**V−CONH$_2$ (**21**)

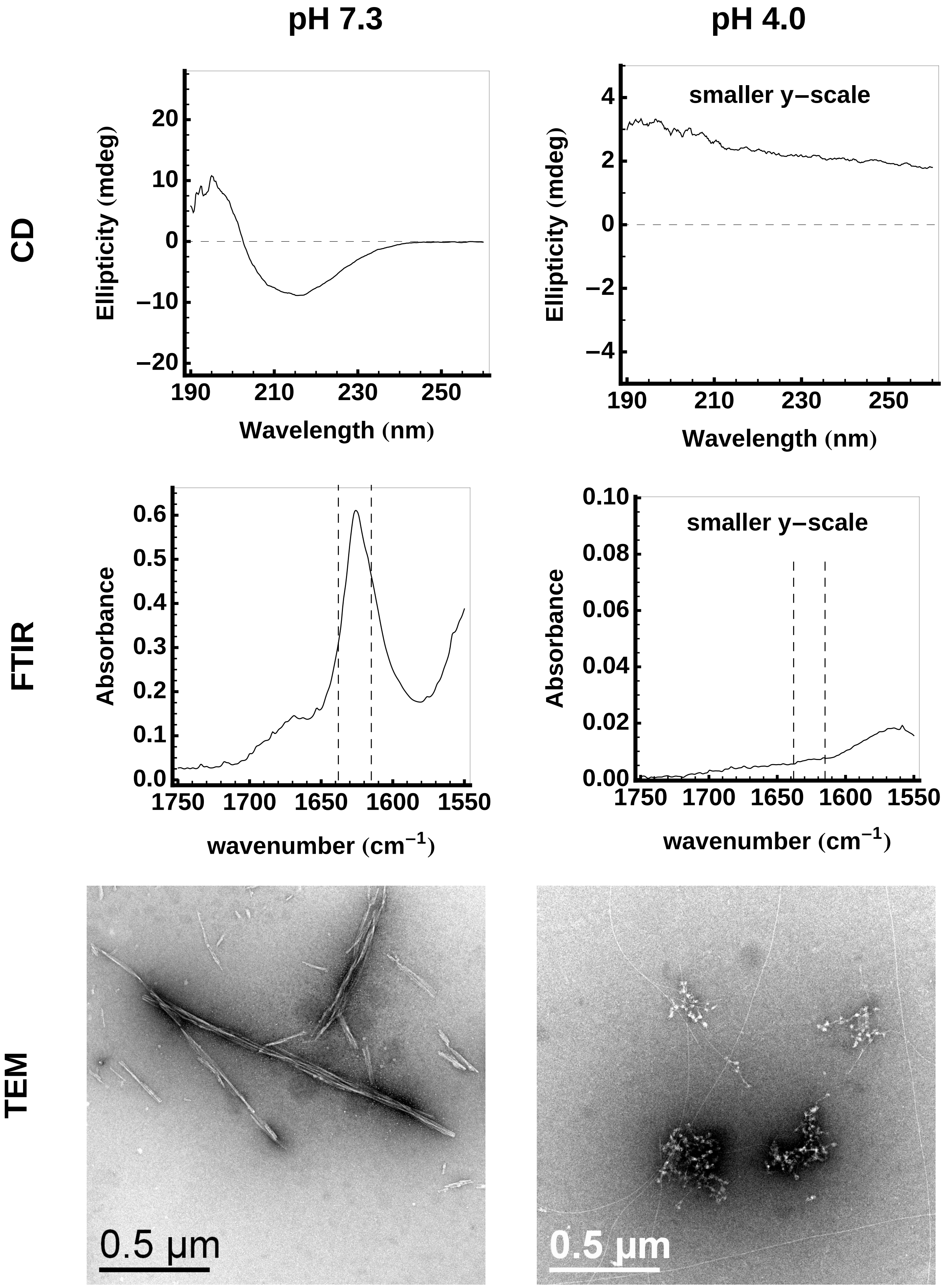

Ac−YV**H**V**D**V**H**V−$CONH_2$ (**22**)

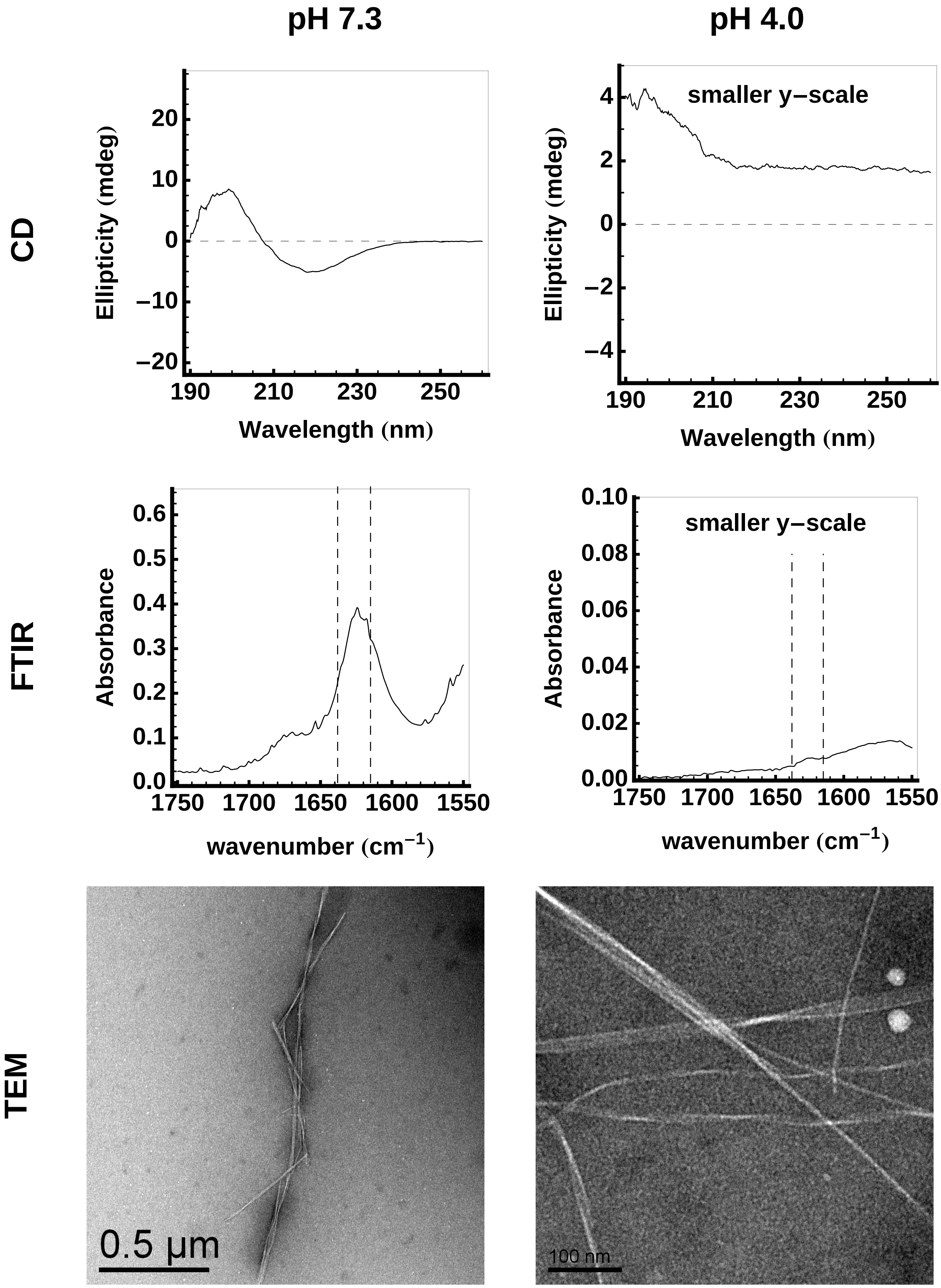

Ac−YV**D**V**H**V**H**V−CONH$_2$ (**23**)

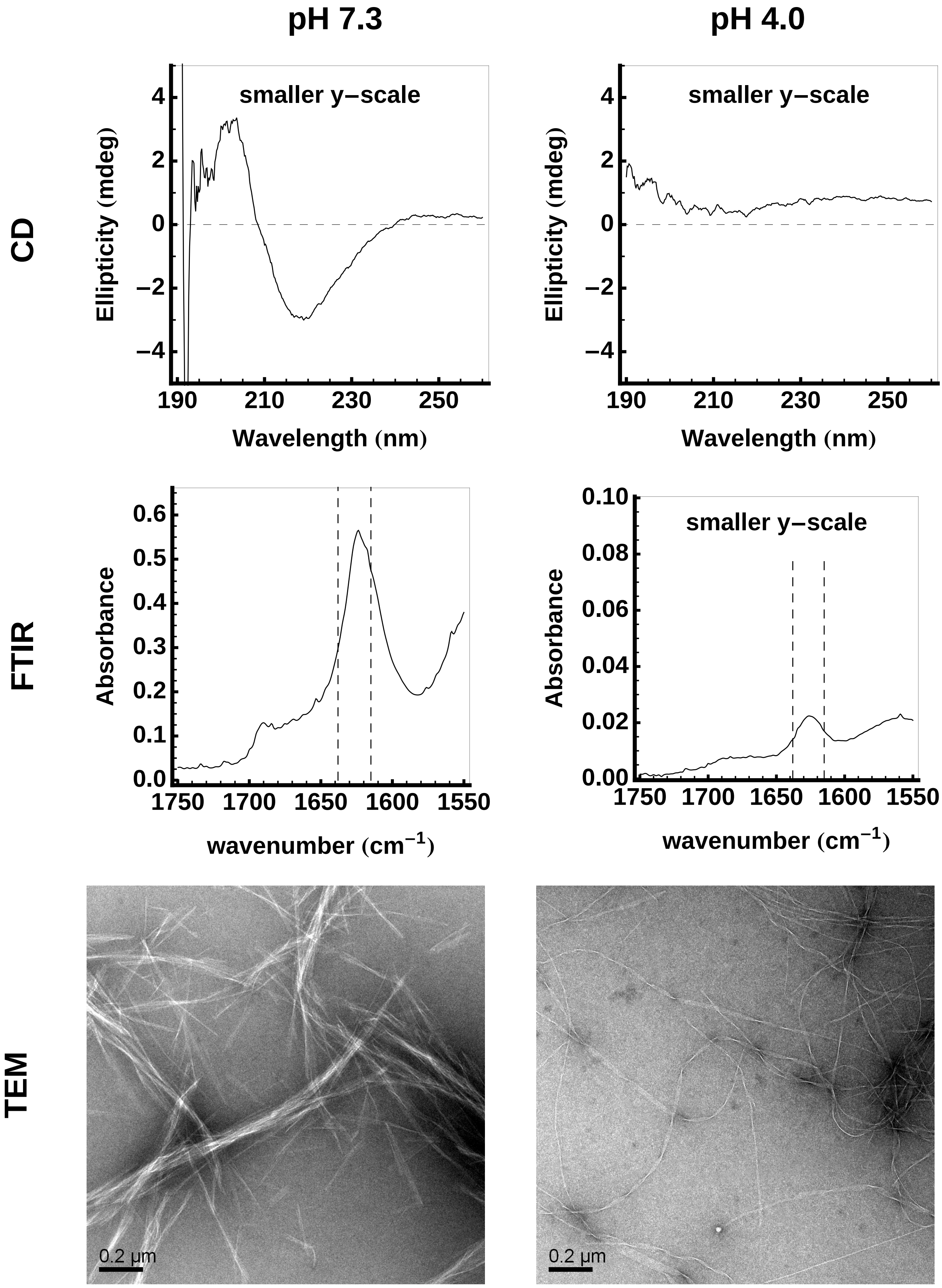

Ac−YV**D**V**D**V**H**V−CONH$_2$ (**24**)

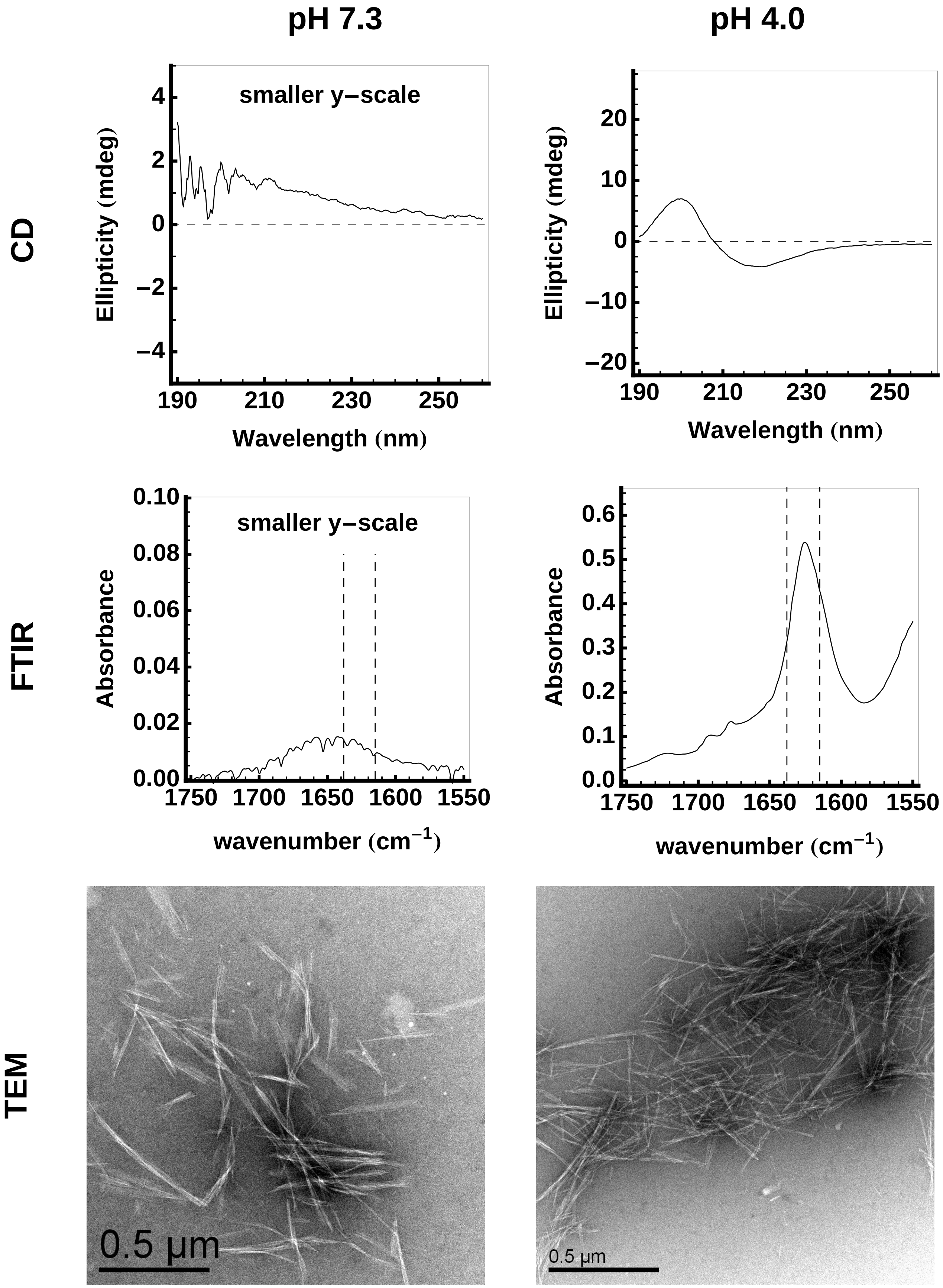

Ac−YV**D**V**H**V**D**V−$CONH_2$ (**25**)

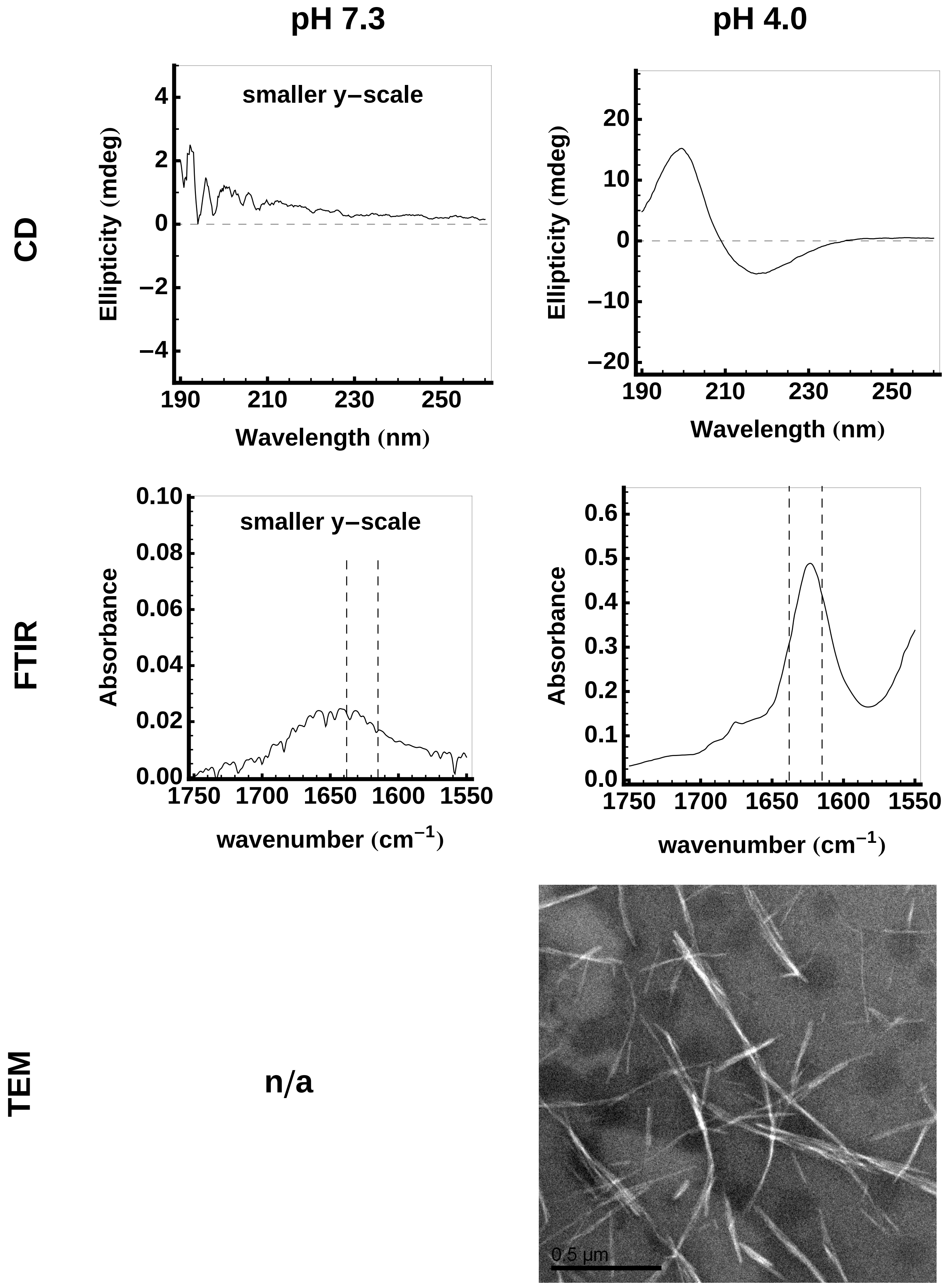

Ac−YV**H**V**D**V**D**V−$CONH_2$ (**26**)

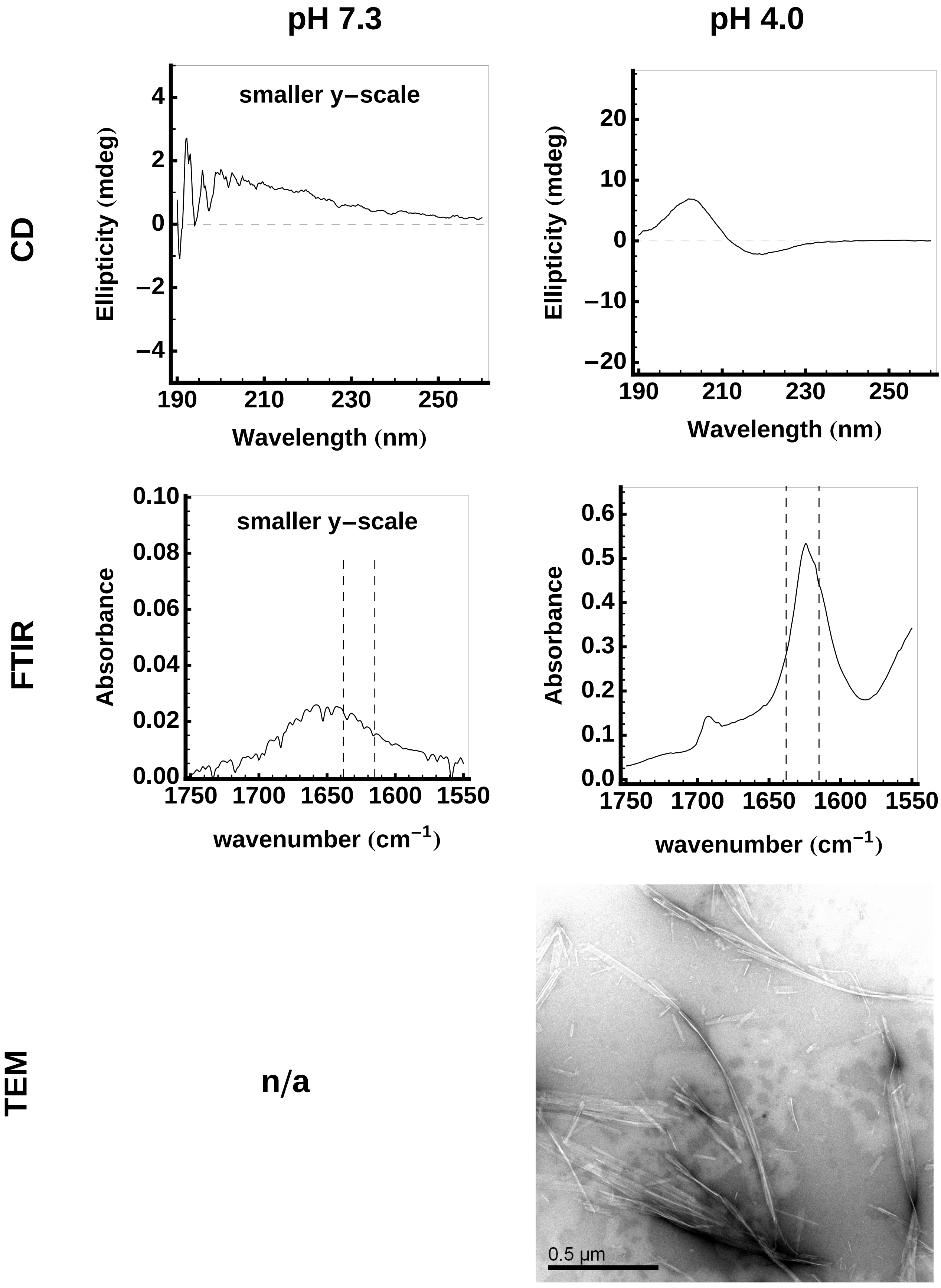

Ac−YV**A**V**H**V**H**V−$CONH_2$ (**27**)

pH 7.3 | **pH 4.0**

CD

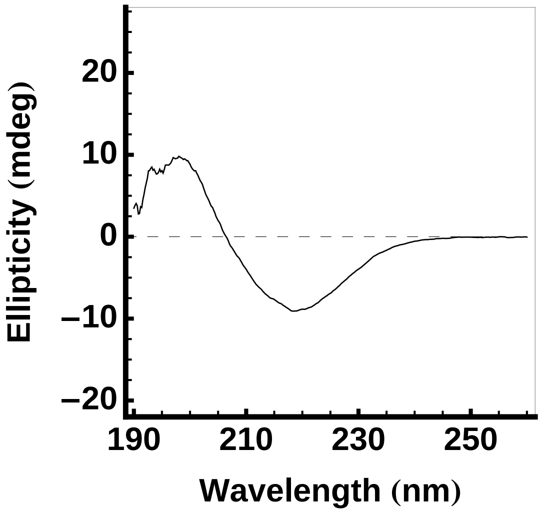

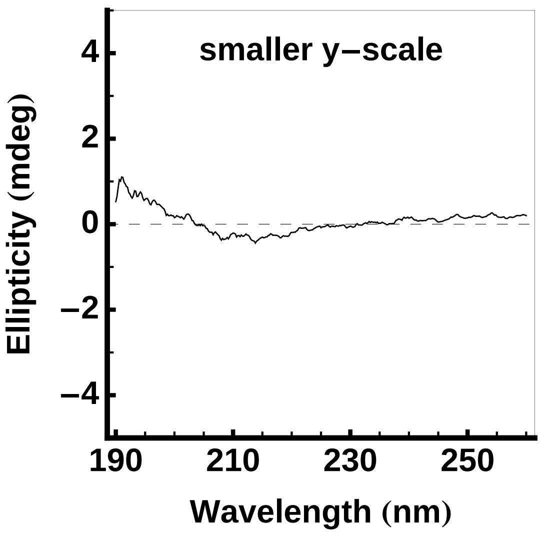

FTIR

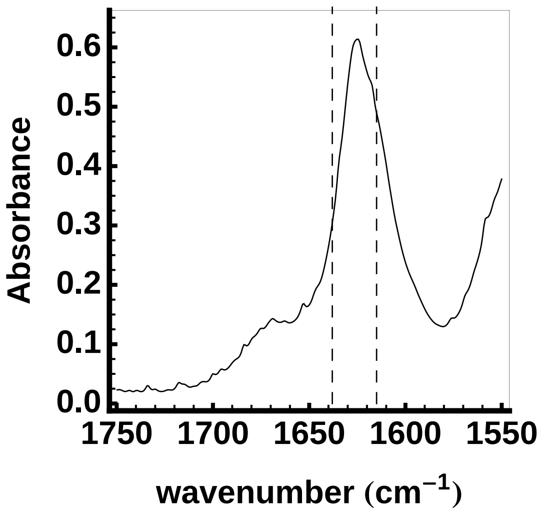

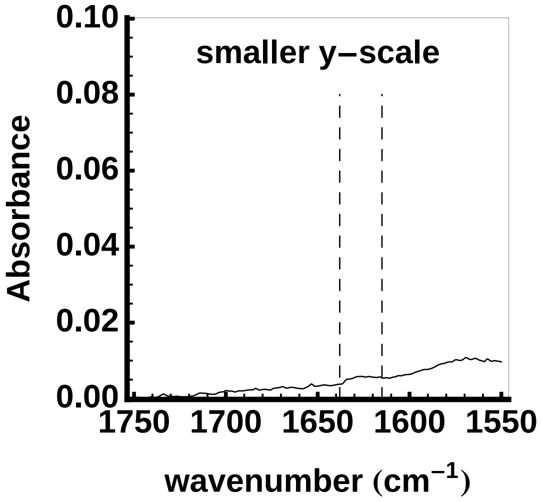

TEM

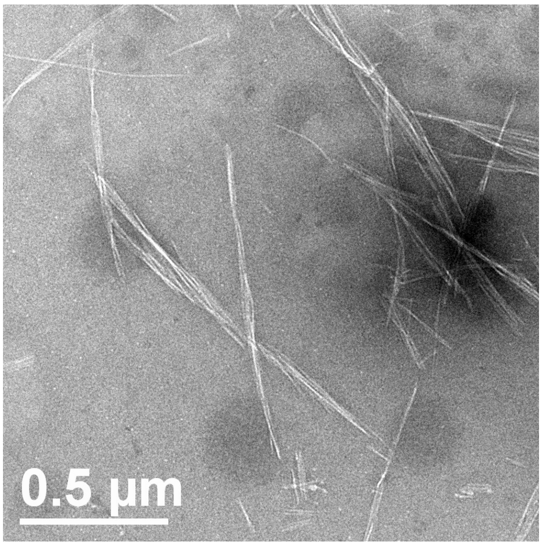

n/a

Ac−YV**H**V**A**V**H**V−$CONH_2$ (**28**)

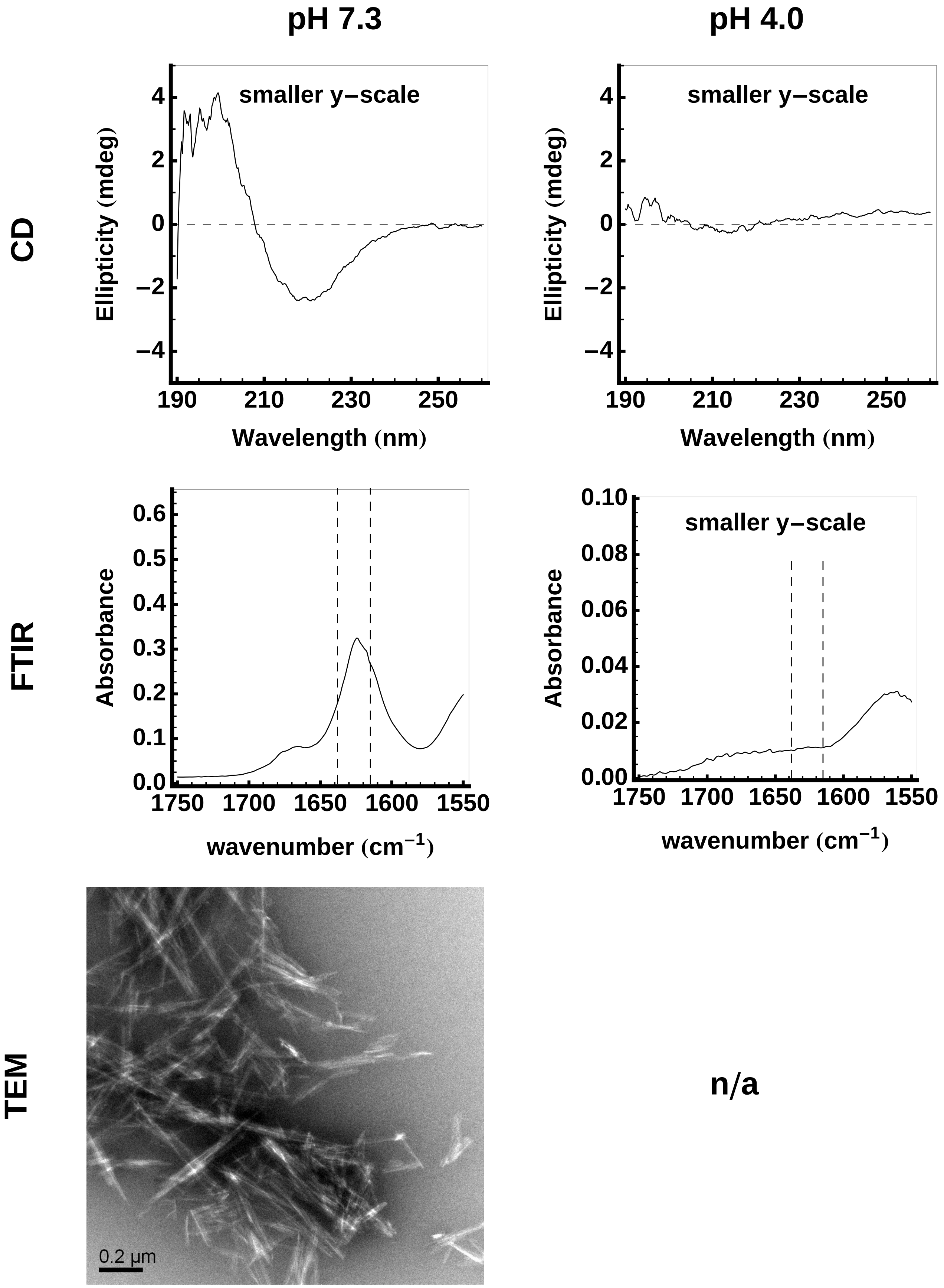

Ac−YV**D**V**H**V**A**V−CONH$_2$ (**29**)

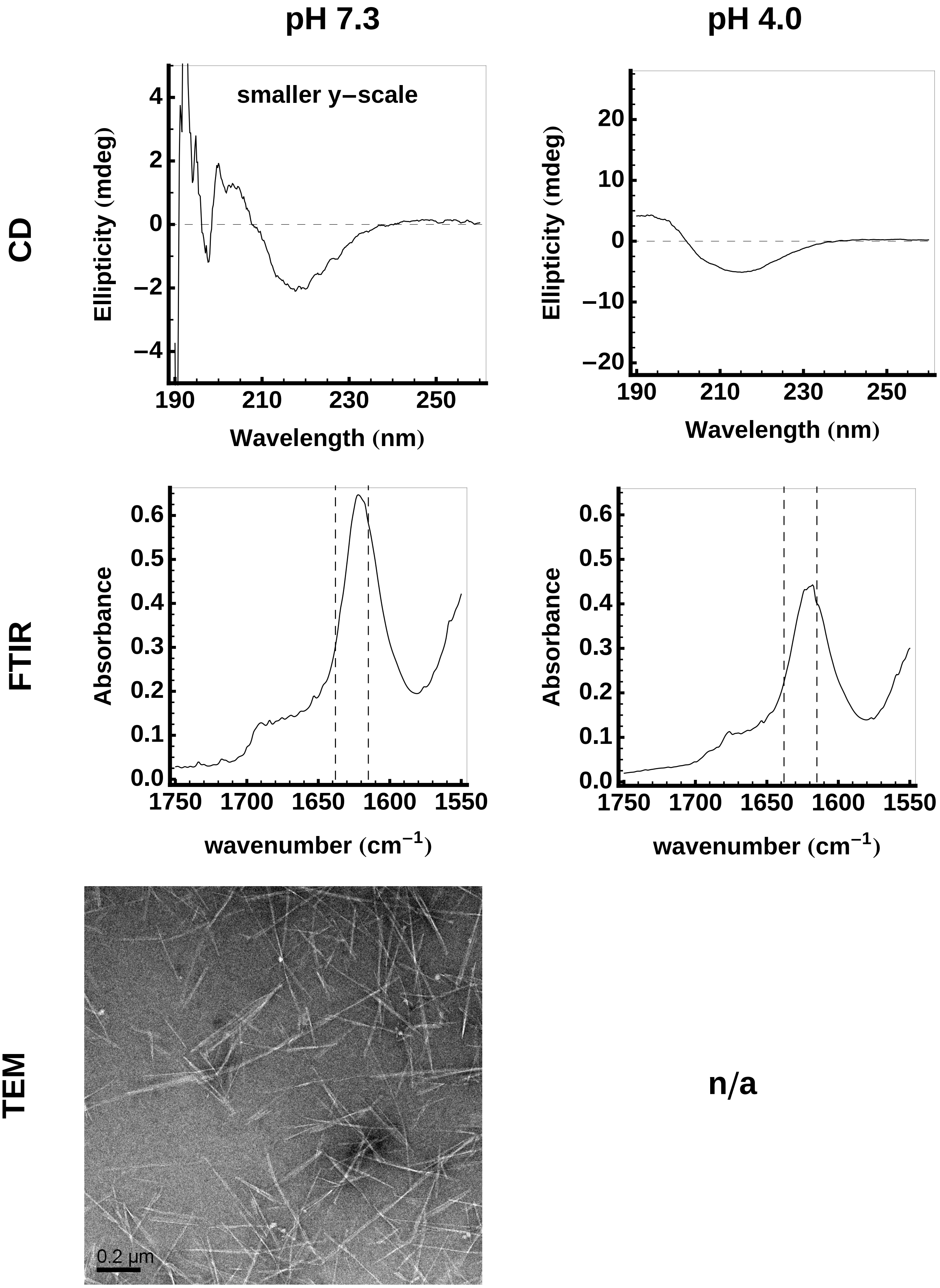

Ac−YV**A**V**D**V**H**V−$CONH_2$ (**30**)

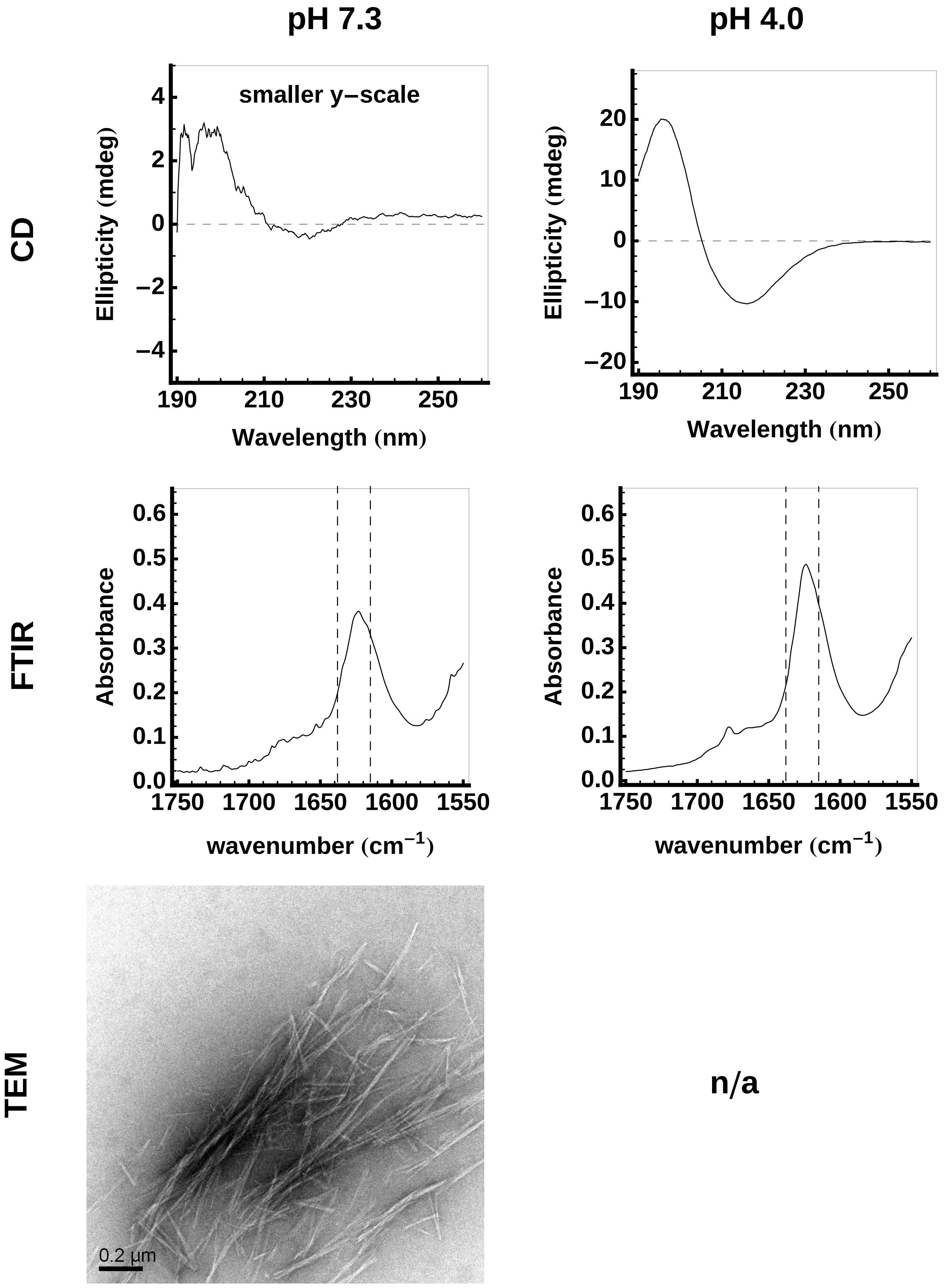

Ac-YV**H**V**D**V**A**V-$CONH_2$ (**31**)

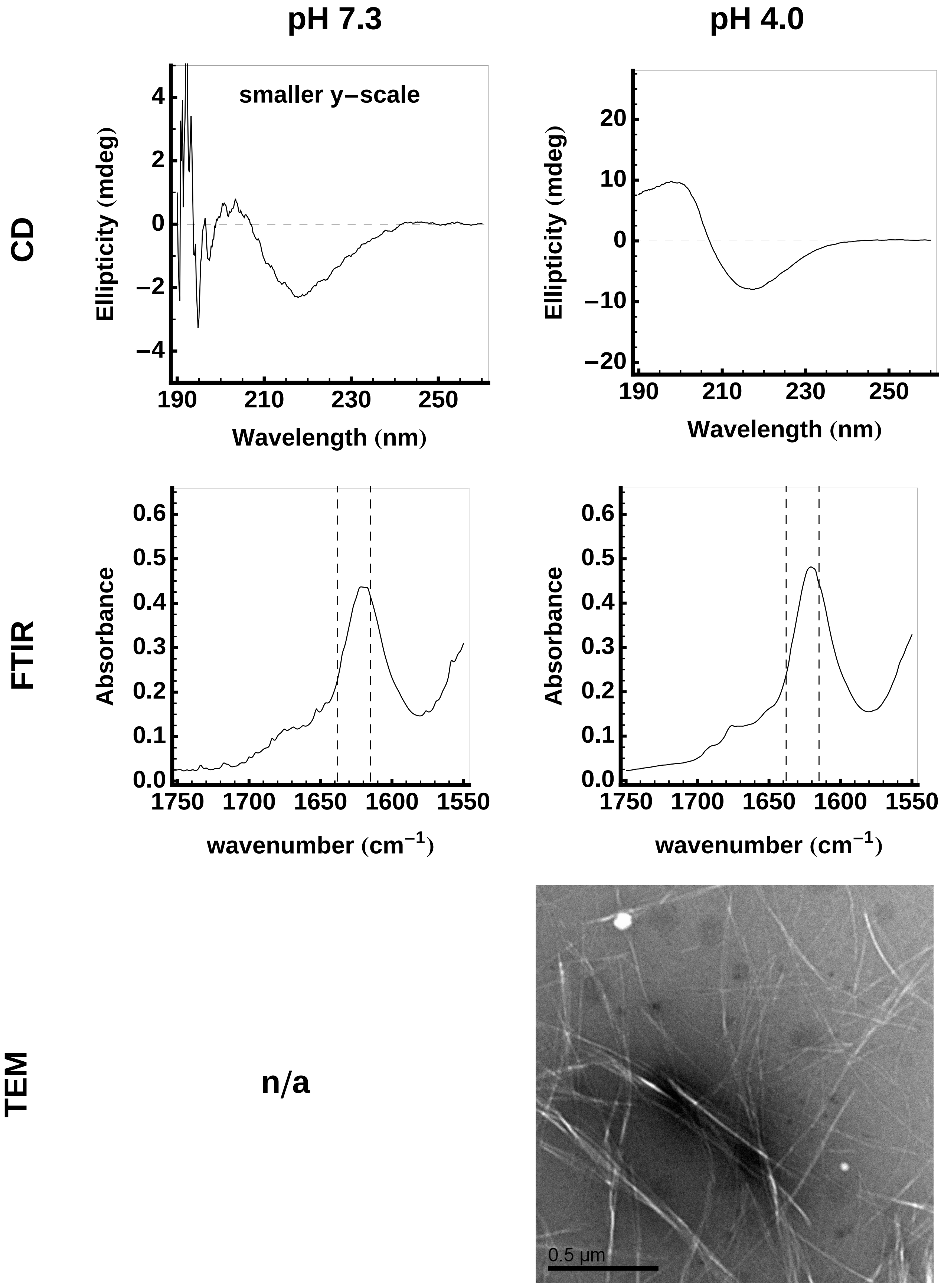

Ac-YV**H**V**A**V**D**V-CONH$_2$ (**32**)

pH 7.3 | **pH 4.0**

CD

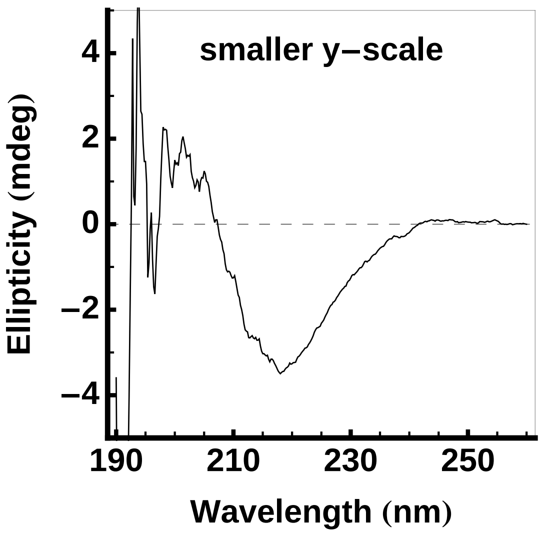

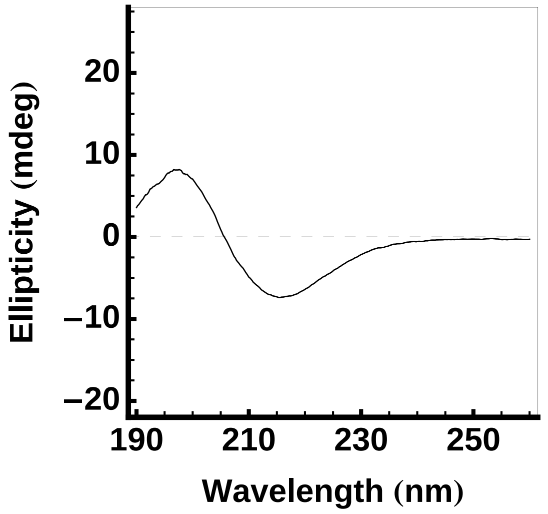

FTIR

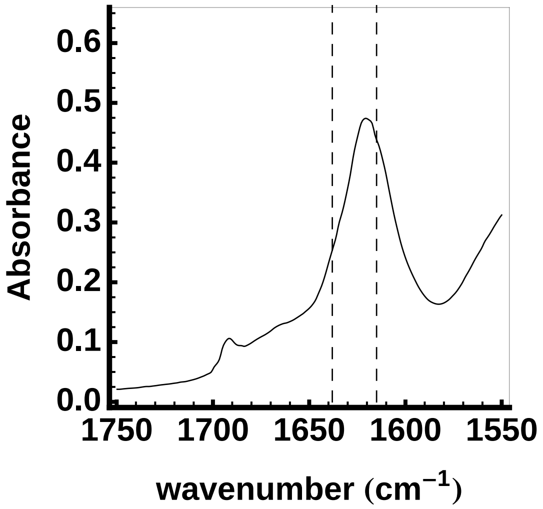

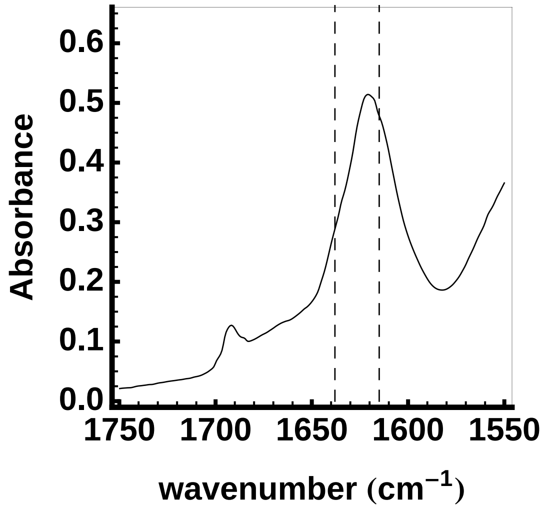

TEM

n/a

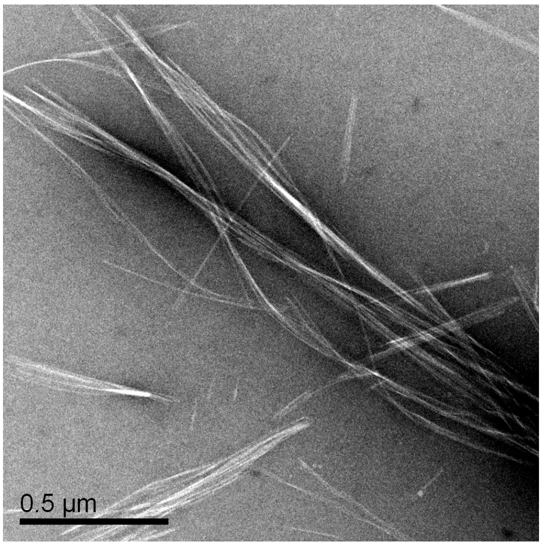

Ac–YV**A**V**H**V**D**V–CONH$_2$ (**33**)

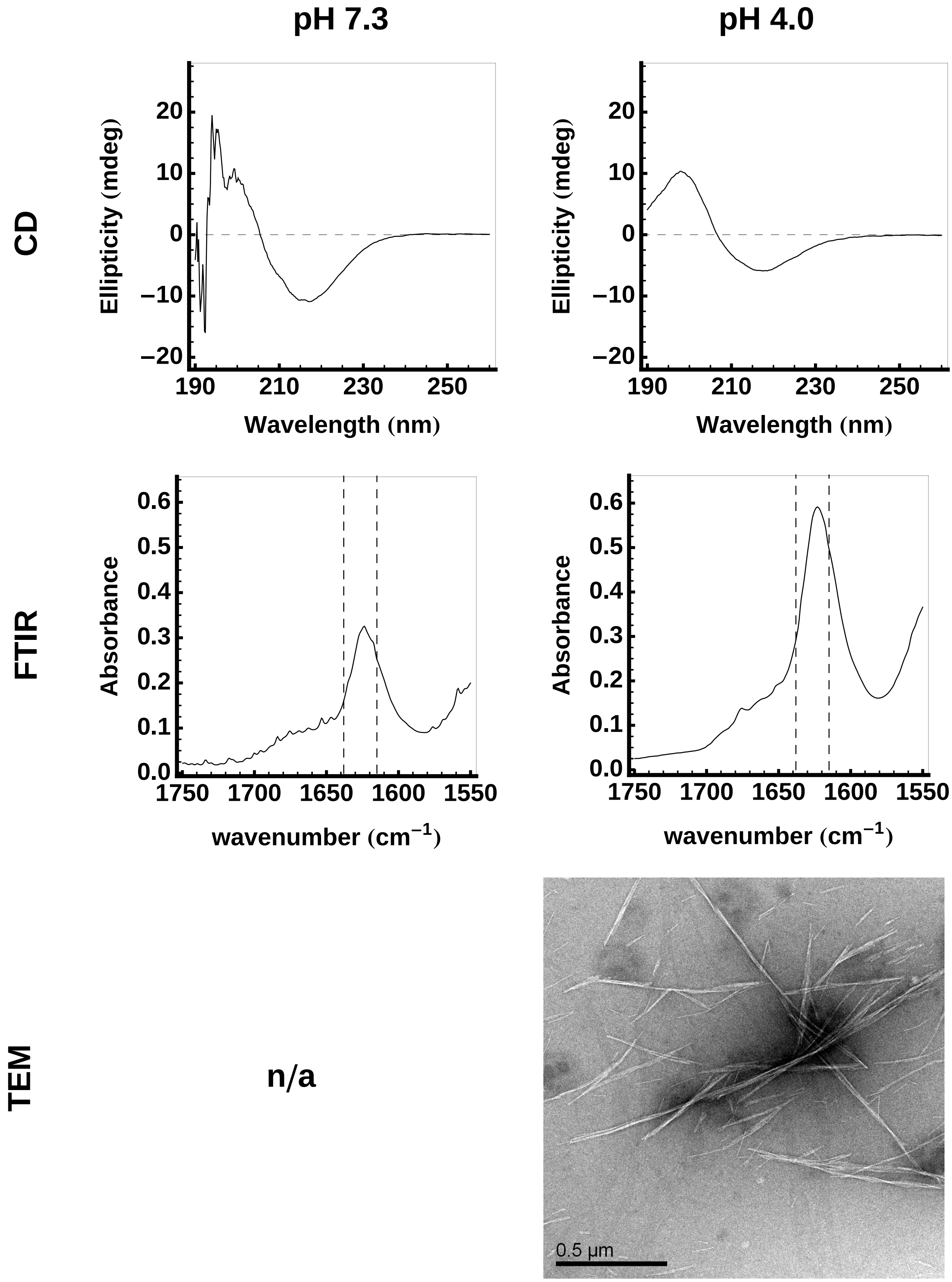

Table B: Amyloid growth and measurement conditions.

	Growth = Measurement[1]			
	Divalent[2]	pH[3]	T (°C)	Additive
1	-	7.3	20	-
2	$ZnCl_2$	7.3	20	-
3	$MgCl_2$	7.3	20	-
4	$CoCl_2$	7.3	20	-
5	$CuCl_2$	7.3	20	-
6	$NiCl_2$	7.3	20	-
7	EDTA	7.3	20	-
8	-	4.0	20	-
9	$ZnCl_2$	4.0	20	-
10	-	2.2	20	-
11	-	7.3	4	-
12	$ZnCl_2$	7.3	4	-
13	-	7.3	20	75 % *i*PrOH
14	-	7.3	20	64 % CH_3CN
15	-	7.3	20	50 % EtOH
16	-	7.3	20	90 % EtOH
17	$ZnCl_2$	7.3	20	75 % *i*PrOH
18	$ZnCl_2$	7.3	20	64 % CH_3CN
19	$ZnCl_2$	7.3	20	50 % EtOH
20	$ZnCl_2$	7.3	20	90 % EtOH
21	-	7.3	20	0.5 M NaCl
22	-	7.3	20	4.75 M NaCl
23	-	7.3	20	0.2 M $MgCl_2$
24	-	7.3	20	2 M $MgCl_2$
25	$ZnCl_2$	7.3	20	0.5 M NaCl
26	$ZnCl_2$	7.3	20	4.75 M NaCl
27	$ZnCl_2$	7.3	20	0.2 M $MgCl_2$
28	$ZnCl_2$	7.3	20	2 M $MgCl_2$
29	-	7.3	20	1 M Na_2SO_4
30	$ZnCl_2$	7.3	20	1 M Na_2SO_4
31	-	4.0	20	2 M $(NH_4)_2SO_4$
32	-	2.2	20	2 M $(NH_4)_2SO_4$

	Growth ≠ Measurement[1]			
	Divalent	pH	T (°C)	Additive
33	$ZnCl_2$	7.3	95	-
34	-	7.3	20	0.5 M NaCl
35	-	7.3	20	4.75 M NaCl
36	-	7.3	20	0.2 M $MgCl_2$
37	-	7.3	20	2 M $MgCl_2$
38	$ZnCl_2$	7.3	20	0.5 M NaCl
39	$ZnCl_2$	7.3	20	4.75 M NaCl
40	$ZnCl_2$	7.3	20	0.2 M $MgCl_2$
41	$ZnCl_2$	7.3	20	2 M $MgCl_2$
42	$ZnCl_2$	7.3	20	75 % *i*PrOH
43	$ZnCl_2$	7.3	20	64 % CH_3CN
44	$ZnCl_2$	7.3	20	50 % EtOH
45	$ZnCl_2$	7.3	20	90 % EtOH
46	$ZnCl_2$	7.3	20	50 % DMSO

1. The conditions for fibrillization and 4NPA assay are the same for 1-32 except that the 4NPA assay was always at 30 °C and the divalent concentration was one-fourth as high. For condition 33, the sample was heated to 95 °C for 1 hour prior to the 4NPA assay and for 34-46 the samples were fibrillized in condition 1 or 2 and then transferred to the new conditions with additive just for the 4NPA assay.
2. Divalent concentration is 1 mM for growth and 250 μM for 4NPA assay.
3. Grown and measured in 50 mM buffer: HEPES at pH 7.3, NaOAc at pH 4.0, or phthalate at pH 2.2.

Figure A: Binary peptide mixtures.

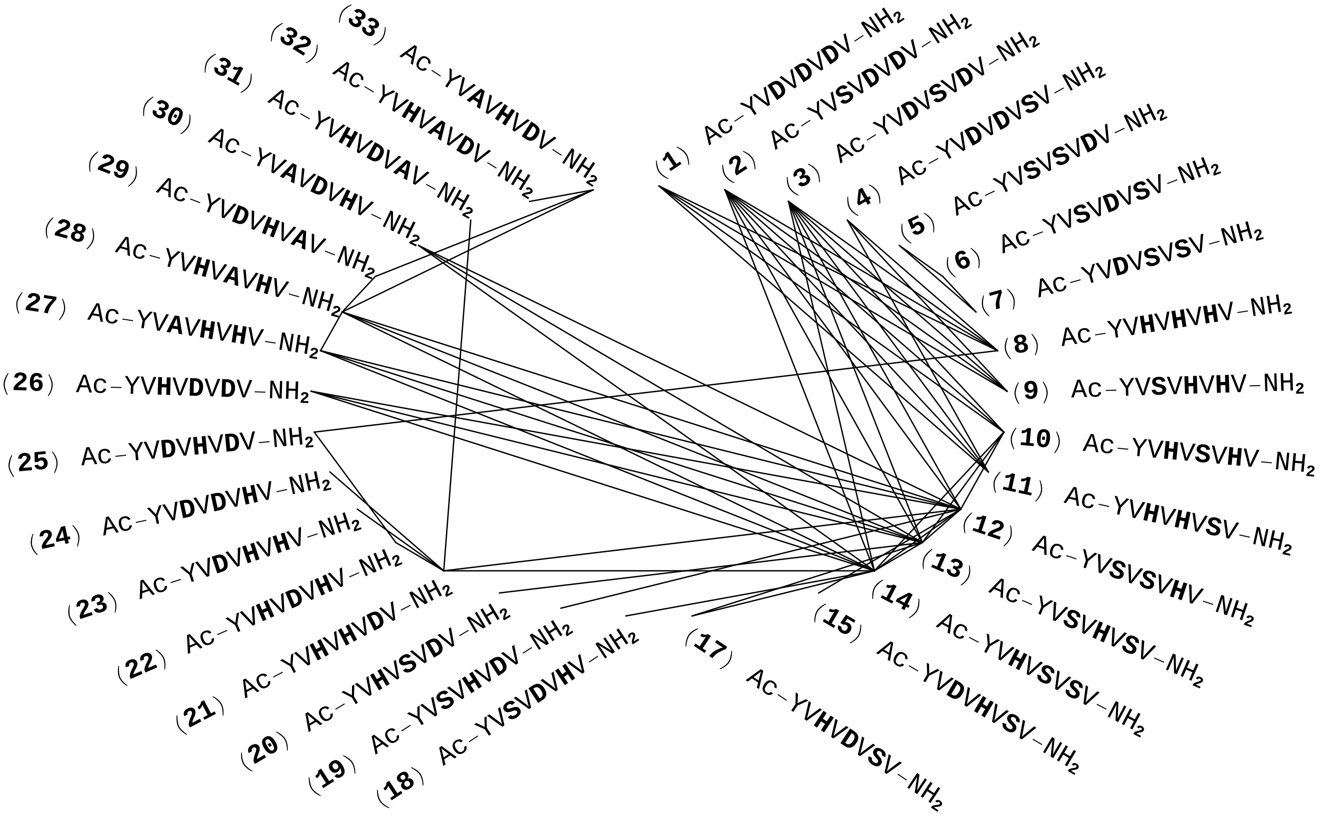

A line connecting the two peptides represents each of the 61 combinations.

Figure B:

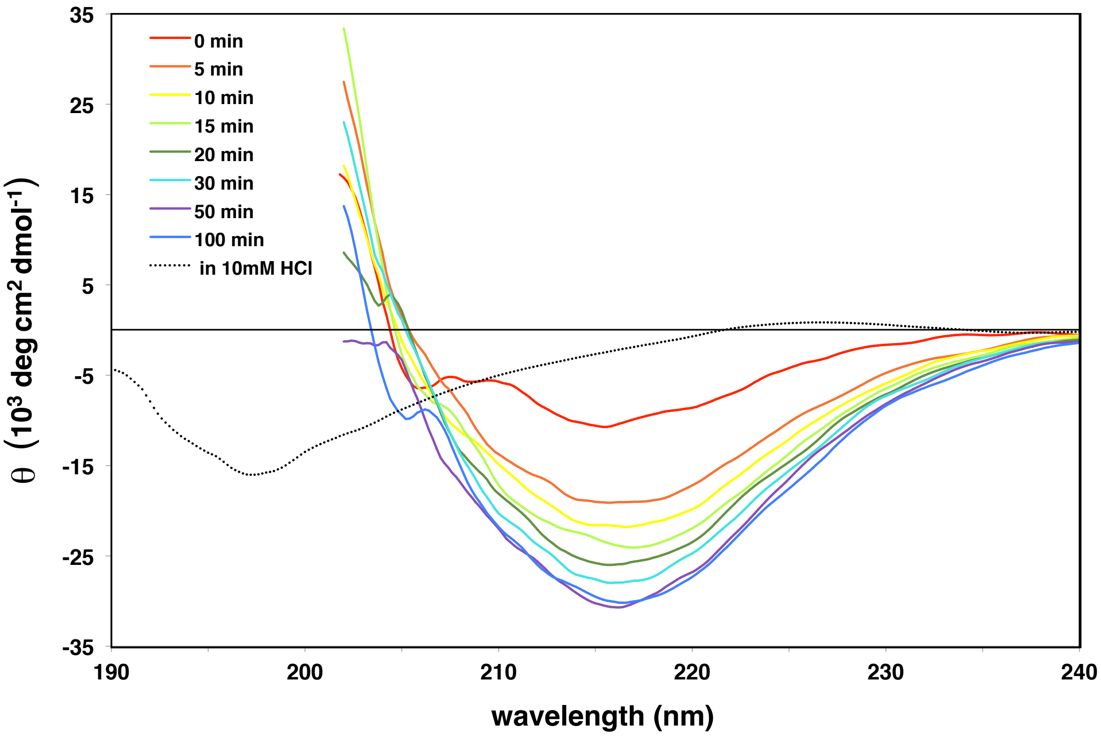

Appearance of β-structure for (**35**) at neutral pH. A 200 μM stock of **35** in 10 mM HCl was diluted to a final concentration of 50 μM into 10 mM HEPES pH 7.3 with 0.5 mM $ZnCl_2$ and the CD spectrum was measured every 5 min. The selected spectra (plotted in rainbow colors) show that the degree of β-structure increases over the first hour of aggregation. The spectrum of 50 μM **35** in 10 mM HCl (dotted line) is time-stable and appears to be mostly random coil.

Figure C:

a

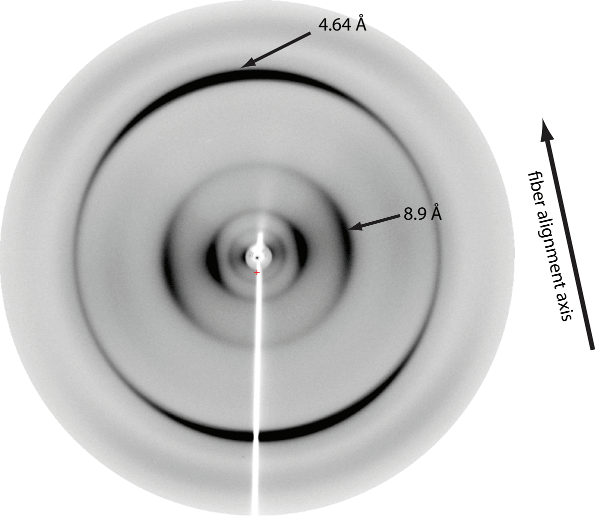

Ac–YV**S**V**D**V**D**V–CONH$_2$ (**2**)

b

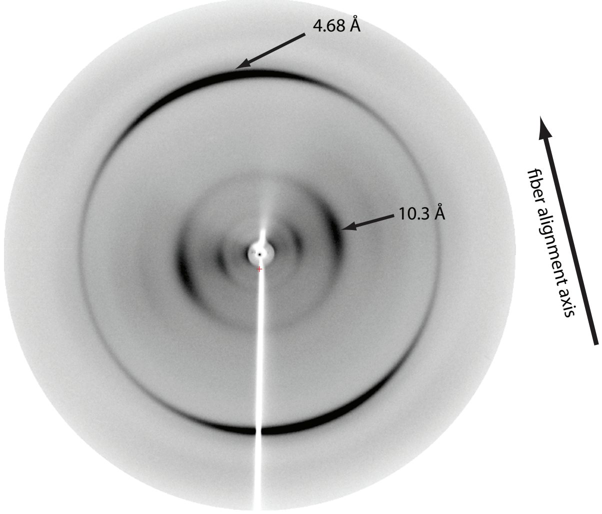

Ac–YV**H**V**H**V**H**V–CONH$_2$ (**8**)

c

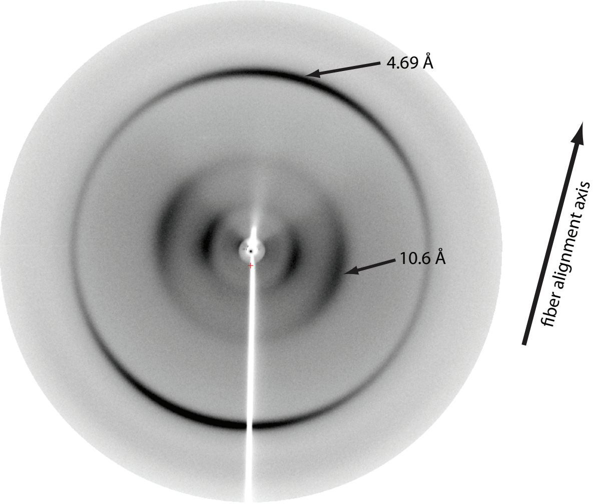

Ac–YV**S**V**S**V**H**V–CONH$_2$ (**12**)

d

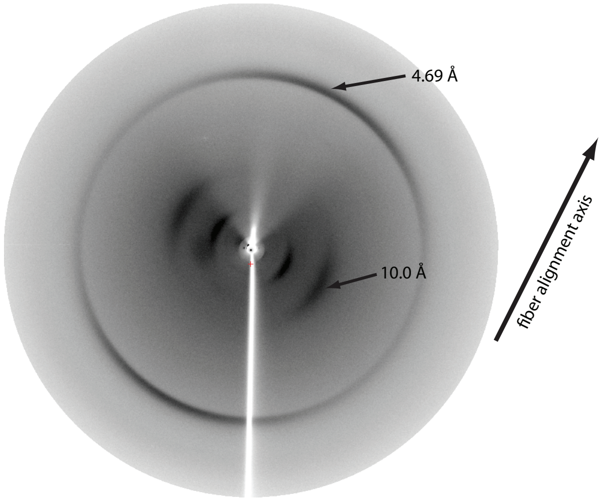

co-aggregate

X-ray diffraction images from aligned fibrils. The peptides **8** and **12** were fibrillized at pH 7.3, washed once in water, and then aligned by drying them between the ends of two glass rods. The peptide **2** in **a** and the co-aggregate of **12** and **21** (Ac–YV**H**V**H**V**D**V–CONH$_2$) in **d** were fibrillized at pH 4 and then washed once in 10 mM NaOAc pH 4 before being aligned by drying.

Figure D:

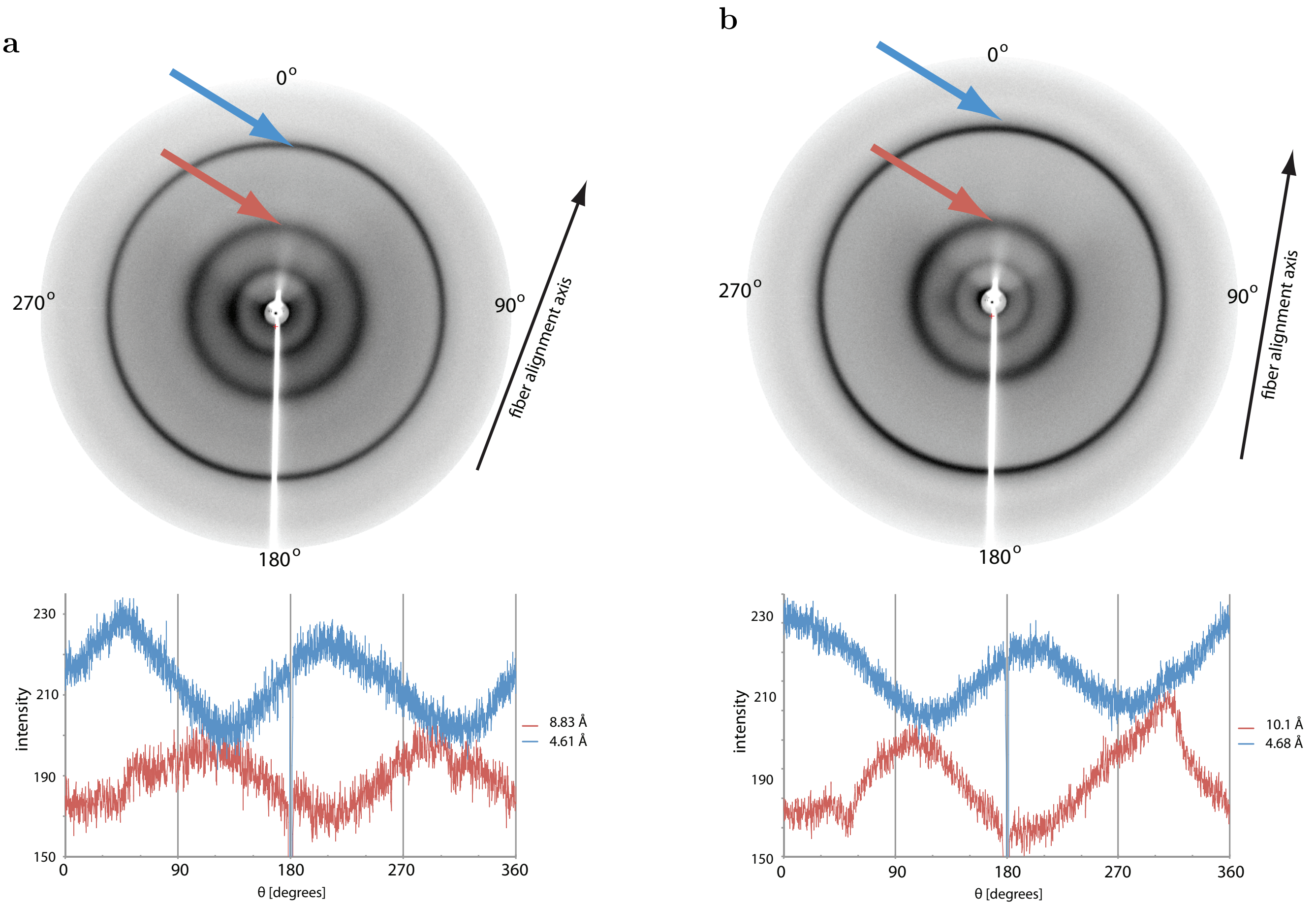

`Ac-YV`**`H`**`V`**`S`**`V`**`S`**`V-CONH`$_2$ (**14**) `Ac-YV`**`A`**`V`**`H`**`V`**`H`**`V-CONH`$_2$ (**27**)

X-ray diffraction images from aligned fibrils with the plot of angular intensity. The fibrils were aligned similarly as in Figure C in S1 File. The diffraction pattern for fibrils of **14** and **27** at pH 7.3 were less clearly cross-β, possibly due to poor alignment. However, the cross-β pattern was present and is more easily visualized in the angular intensity distribution for the two major spacings.

References

[1] Tatulian, S. A. (2013). *Structural characterization of membrane proteins and peptides by FTIR and ATR-FTIR spectroscopy.* Methods in Molecular Biology (Clifton, N.J.), 974(Chapter 9), 177218. `http://doi.org/10.1007/978-1-62703-275-9_9`
